# High Gene Flow With Patterns of Asymmetric Connectivity and Adaptive Divergence in the New Zealand Southern Rock Lobster, *Jasus edwardsii* (Hutton, 1875)

**DOI:** 10.1111/eva.70233

**Published:** 2026-04-07

**Authors:** Michael J. R. Mendiola, Irina Ilushkina, Jan M. Strugnell, Nick Murphy, James J. Bell, Jonathan P. A. Gardner

**Affiliations:** ^1^ School of Biological Sciences Victoria University of Wellington, Kelburn Wellington New Zealand; ^2^ Centre for Sustainable Tropical Fisheries and Aquaculture James Cook University Townsville Australia; ^3^ Department of Ecological, Animal and Plant Science La Trobe University Melbourne Victoria Australia

**Keywords:** asymmetrical gene flow, environmental variation, fisheries management, population genetic structure, population genomics, seascape genomics

## Abstract

Understanding patterns of connectivity and adaptive divergence is crucial for supporting conservation and sustainable management of harvested species. This study utilised single nucleotide polymorphisms (SNPs) to investigate spatially explicit patterns of genetic structure, gene flow and adaptive divergence in the commercially important southern rock lobster *Jasus edwardsii* across New Zealand (NZ), a species with a 2‐year pelagic larval duration. Using neutral (1608 SNPs) and outlier (250 SNPs) panels, genetic analyses revealed significant population differentiation between *J. edwardsii* individuals collected from one site in Tasmania (Australia) and those from 22 sites in NZ, supporting earlier findings indicating substantial genetic variation between the two stocks. Within NZ, neutral markers revealed a high degree of genetic connectivity of *J. edwardsii* , but also subtle genetic differentiation of one northern site only. In contrast, analysis of outlier markers identified three genetically differentiated regions within NZ: the Northeast (NE), Northwest (NW) and Southern (S) groups. Migration models indicated bidirectional but asymmetrical gene flow amongst these groups (S → NW → NE), consistent with larval dispersal and settlement models. Patterns of genetic structure at the outlier loci were strongly correlated with the environmental variables ‘carbonate concentration’, ‘roughness at the seafloor’ and ‘sea surface temperature’ across NZ, which may contribute to differential settlement and survival of *J. edwardsii* . These findings demonstrate complex patterns of connectivity and the influence of environment factors on genetic structure and local genetic adaptative divergence of *J. edwardsii* despite its very high dispersal potential. The population genomic and connectivity findings support the current spatially explicit management regime of the rock lobster fishery in New Zealand, and the seascape genomic findings may be of value to the fishery in responding to new or enhanced patterns of environmental variability driven by climate change or to the development of a new aquaculture industry.

## 1 Introduction

Understanding patterns of population connectivity and spatially explicit genetic differentiation is critical for the conservation and sustainable management of harvested species (Kough et al. 2013; Burgess et al. 2014). For most benthic marine species, adult life stages exhibit relatively restricted patterns of movement (Barnes and Hughes 1999) and population connectivity is generally achieved through planktonic larval dispersal, with larvae often travelling large distances (Kritzer and Sale 2006). Because pelagic larval duration (PLD) varies considerably amongst species, from hours to years (Strathmann and Strathmann 2007; Shanks 2009), different species exhibit different routes and rates of connectivity (Puebla et al. 2012; Benestan et al. 2015; Poćwierz‐Kotus et al. 2015). Traditionally, PLD was thought to correlate with dispersal distance due to the large numbers of larvae, ocean current patterns and a lack of obvious barriers in the sea to larval transport (Shanks et al. 2003; Pascual et al. 2017) but recent studies have challenged this assumption by describing patterns of self‐recruitment and local‐scale larval retention (Chaput et al. 2023; Quigley et al. 2023; Michie et al. 2024). Gaining knowledge of how patterns of population connectivity in the sea may have originated and may now be maintained remains a major challenge, in particular for species with long (> 3 to 6 months) or very long (> 6 months) PLDs. This knowledge gap has long been recognised (e.g., Palumbi 2003) because information about connectivity can contribute to conservation and management (e.g., fisheries) decisions yet is only infrequently available or considered (Zeng et al. 2020; Holland et al. 2022; Rieder et al. 2025).

Neutral locus diversity is mainly a product of random processes such as mutation and genetic drift (Luikart et al. 2003) whereas adaptive locus diversity is mainly the result of selective processes (Galindo et al. 2009). Thus, neutral loci are most informative for estimates of gene flow and population history whereas adaptive loci are most informative for identifying or estimating selection (Morin et al. 2004). However, while gene flow acts to homogenise gene pools, selection leads to an increase in the allele frequencies of advantageous or adaptive genes (Kirk and Freeland 2011). It is becoming increasingly apparent that environmental gradients in marine systems can influence a species' local recruitment success, its population sizes and may also counteract the effects of gene flow, which results in locally adapted genotypes (e.g., Orsini et al. 2013; D'Aloia et al. 2015), even in species with long PLDs (e.g., Villacorta‐Rath et al. 2016). Population differentiation arising from the combination of selection and gene flow will depend on the strength of the selection pressure and the effectiveness of the gene flow, and will be a function of the population size (smaller populations will be more affected than larger populations). In natural populations, the interplay of genetic drift, gene flow and selection can therefore result in distinct patterns of differentiation in adaptive versus neutral genes (e.g., Freamo et al. 2011; Matala et al. 2014), often with loci under selection having greater power to resolve population differentiation than neutral loci (Wei et al. 2013a; Milano et al. 2014; Villacorta‐Rath et al. 2016).

There are major challenges in linking patterns of genetic variation (both neutral markers and those under selection) and environmental variation in marine systems, particularly as a result of the high temporal variability in environmental factors (Liggins et al. 2013; Rieder et al. 2025). Temperature is one of the most commonly studied drivers of adaptive divergence because it is generally easy to measure (often via satellite data), and this effect has been reported in a number of marine invertebrates, including the New Zealand green‐lipped mussel, *Perna canaliculus* (Wei et al. 2013b), North Atlantic shrimp, *Pandalus borealis* (Jorde et al. 2015; Bourret et al. 2024), orange mud crabs, *Scylla olivacea* (Mendiola and Ravago‐Gotanco 2021) and the American lobster, *Homarus americanus* (Benestan et al. 2015). However, the increasing availability of more complex and spatially explicit environmental datasets (e.g., a new national environmental dataset for New Zealand—Table S1) means that it is likely that environmental factors, other than just sea surface temperature, may also be shown to explain species‐specific genetic variation. Historically, many seascape genetics studies have employed neutral microsatellite genetic markers for inferring environmental barriers to gene flow that might cause divergence of local populations via genetic drift. However, while neutral marker variation has long been believed to not confer fitness advantages (Slatkin 1987—but see Kardos et al. 2023), variation of non‐neutral (outlier or adaptive) markers might have a direct effect on fitness and the survival of the individuals under selection. In the absence of geographical or topographic barriers to gene flow in the marine environment, seascape analysis of neutral markers can provide insight into biological barriers or environmental selection that consistently reduce or counteract gene flow (Wei et al. 2013b; Benestan et al. 2016; Silva and Gardner 2016; Zeng et al. 2020). However, while neutral markers generally reflect long‐term population connectivity patterns (i.e., population history), markers under adaptive selection provide information about more recent environmental and demographic processes (Holderegger et al. 2006). If divergence of markers under selection across habitats can be linked to environmental factors, it may ultimately be possible to identify candidate genes on which selection is acting and thereby provide insights into genotype‐environment interactions (Schmidt et al. 2008).

The New Zealand marine environment ranges from subtropical to subantarctic waters and, as such, conditions vary along multiple environmental gradients at multiple spatial scales. This environmental gradient provides a range of selective conditions that might explain patterns of adaptive population differentiation in species occurring throughout this range. One such species is *Jasus edwardsii* (Hutton, 1875), a spiny lobster belonging to the family Palinuridae that inhabits rocky reefs between 1 and 200 m deep along the coastlines of southern Australia and New Zealand (Fisheries New Zealand 2023). In New Zealand, its range extends from the Three Kings Islands in the north (34° S, 172° E) to the Auckland Islands in the south (51° S, 166° E), and to the Chatham Islands in the east (44° S, 176° W) (Kensler 1967). *J. edwardsii* has a complex life cycle that is largely but perhaps not yet fully understood: adults inhabit rocky reefs where they spawn, and the first development stage, the naupliosoma, moults into a phyllosoma within several hours of being released. The phyllosoma drift with offshore currents for 12–24 months, where they have some ability to move vertically in the water column (Bradford et al. 2005), before they return to inshore waters, where they metamorphose into a post‐larva, known as a puerulus. Pueruli have been shown to have sufficient energy reserves to be able to swim actively up to 200 km from the edge of the continental shelf to coastal reefs (Jeffs et al. 2001), so once they reach the continental shelf from the open ocean environment their movements to inshore reefs are deliberate. Pueruli can respond to a variety of cues, likely to orient toward their settlement grounds, including underwater sound, water chemistry, eddy‐chemotaxis, salinity and magnetic fields (Cobb 1997; Jeffs et al. 2005; Hinojosa et al. 2018). The puerulus moults into a juvenile a few days after settlement (Booth and Phillips 1994). Once settled, rock lobster movements generally do not exceed 5 km (Booth 1997), meaning that most demographic and genetic connectivity occurs as a result of the pelagic life stages, where drifting larvae may travel 1000s km. The wide geographical distribution of the lobster across NZ waters means they inhabit a number of environmentally distinct locations that may create selective pressure sufficient to counteract the high levels of expected gene flow for a PLD of up to 2 years.

**FIGURE 1:**
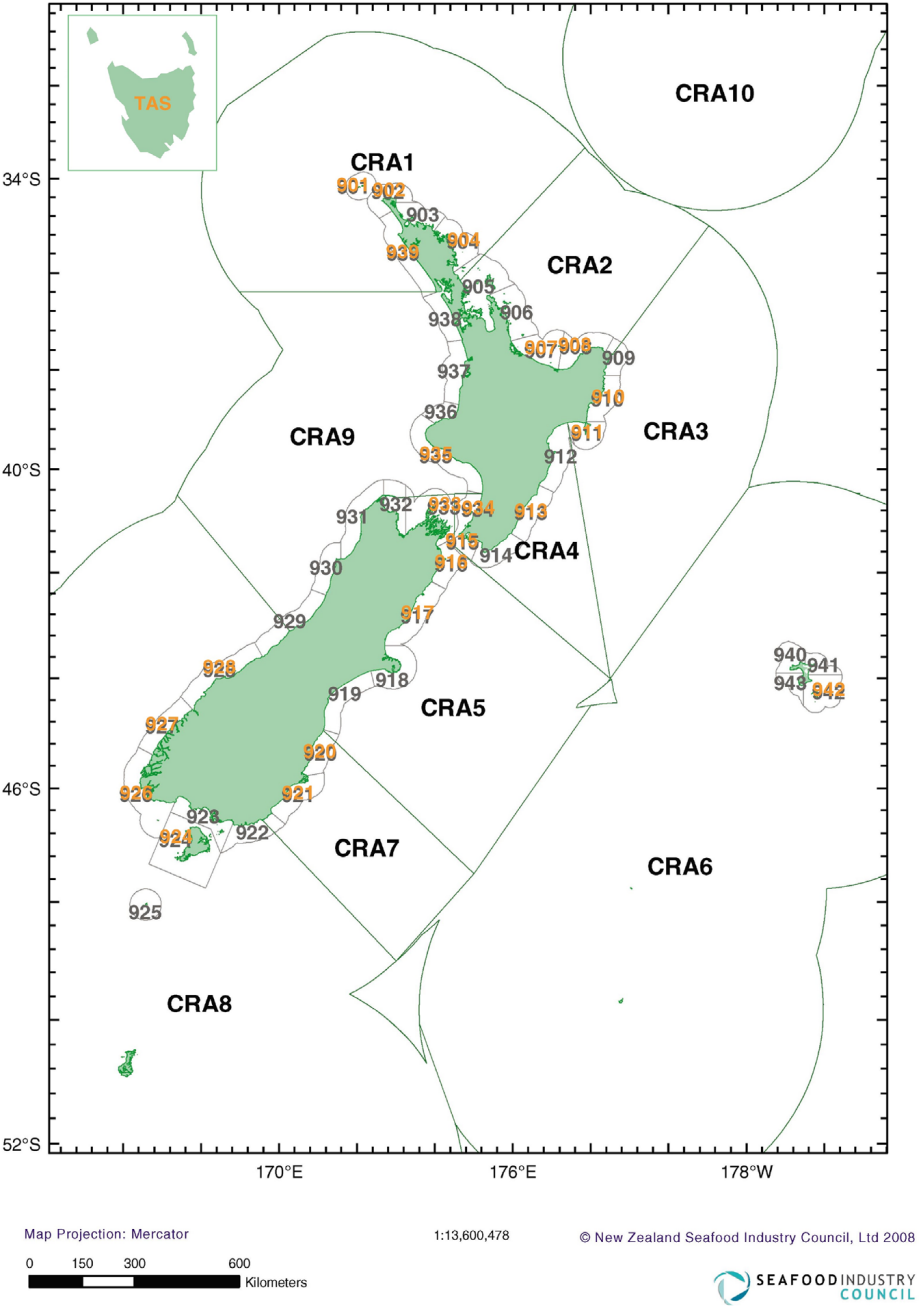
Map of the New Zealand rock lobster (locally known as ‘crayfish’ abbreviated as CRA) quota management areas (QMAs) CRA1‐10. The highlighted regions (numbers in yellow) represent statistical areas within QMAs where samples were collected (collection sites), along with a sample from Tasmania, Australia (TAS). This map was adapted and modified from Starr (2010).

Modelling of *J. edwardsii* settlement patterns (Chiswell and Booth 2008, 2017) identified four main areas of dispersal within NZ. Populations in the far North (CRA1—Figure 1) receive most of their settlers from the West coast of NZ (CRA8 and CRA9—Figure 1) and supply larvae to the populations along the East coast (CRA3 and CRA4—Figure 1). In turn, the East coast region entrains a large number of locally produced settlers in the Wairarapa Eddy (CRA4—Figure 1) and contributes moderately to the larval pool in the South of NZ (CRA5, CRA7 abd CRA8—Figure 1). Lastly, high levels of self‐recruitment were predicted by the model to occur in the South Island (CRA7 and CRA8—Figure 1). A number of genetic tools have been used to estimate *J. edwardsii* genetic population connectivity, and while early studies (using low resolution markers) found little evidence of differentiation across the species' range (Smith et al. 1980; Booth et al. 1990; Ovenden et al. 1992). Further studies (Morgan et al. 2013; Thomas and Bell 2013) described significant differentiation between Australian and New Zealand lobster stocks using microsatellite markers, and some differentiation within New Zealand. More recently, differentiation between Australian and New Zealand lobsters was confirmed using a panel of SNPs (Villacorta‐Rath et al. 2016), with the SNPs also revealing chaotic genetic patchiness and evidence of post‐settlement selection in Australian populations (Villacorta‐Rath et al. 2018). However, to date, no studies have investigated the impact of environmental gradients on driving signatures of genetic differentiation in *J. edwardsii* .

Environmental factors vary across the range of *J. edwardsii* , particularly from south to north, such as coastal sea surface temperature (10°C–16.5°C; Chiswell and Grant 2018) and salinity (34.2–35.6 psu; Leathwick et al. 2012). Temperature has been reported to affect all life stages of rock lobsters, including the phyllosoma (Bermudes and Ritar 2008; Smith et al. 2002), post‐puerulus (Dubber et al. 2004), juvenile (Crear et al. 2000) and adult (Thomas et al. 2000) stages, making it a strong candidate for causing adaptive divergence. However, in the case of *J. edwardsii* , high levels of larval dispersal (connectivity) may negate the effects of local selection to some extent over generations. This is because the extent of adaptive divergence and local adaptation depends on whether barriers to dispersal are a cause or a consequence of phenotypic differentiation, and evolutionary forces may act in favour or impede these processes (Sotka 2012; Gary et al. 2020). Therefore, this study developed two panels of single nucleotide polymorphism (SNP) markers: one consisting of neutral loci and the other of loci putatively under diversifying selection (referred to as outlier loci). Specifically, this research aimed to: (1) assess the spatially explicit population genetic structure of the southern rock lobster *J. edwardsii* in New Zealand; and (2) identify any correlation between outlier loci and multiple independent environmental variables across New Zealand to identify potential drivers of localised adaptive divergence.

## 2 Materials and Methods

### 2.1 Sampling and DNA Extraction

In total, 615 adult lobsters (individuals) were analysed. Lobsters were collected between July 2015 and March 2016 from 22 New Zealand locations (henceforth sampling sites or sites), including offshore islands (Figure 1; Table 1). In addition, 29 adult lobsters from Tasmania (Australia) were included to serve as a reference group for comparison with previously published results (Morgan et al. 2013; Thomas and Bell 2013; Villacorta‐Rath et al. 2016). Adult male and female lobsters were sampled in equal proportions, where possible, by commercial fishermen using baited pots. A lobster's pereiopod (walking leg) was removed by commercial fishermen and preserved in 96% ethanol until required for DNA extraction. The number of samples per site varied between 14 and 30 (mean ± SD of 27.08 ± 5.69). DNA from preserved samples was extracted using a DNeasy Blood and Tissue kit (Qiagen) following manufacturer's instructions. DNA concentration was quantified using a Qubit 2.0 Fluorometer (Life Technologies) and DNA purity was assessed using a NanoDrop 2000. Gel electrophoresis was used to determine DNA integrity. Only relatively intact (molecular weight ≥ 1000 bp) and pure (260/280 ≥ 1.8) DNA samples were used for ddRADseq library preparation.

**TABLE 1:** Sampling information of 615 *Jasus edwardsii* individuals and genetic diversity plus effective population size (*N*e) estimates for each site.

Site code	Coordinates lat, long	*N*	Neutral panel (1608 SNPs)				Outlier panel (250 SNPs)				*N*_e_
			*A*_R_	*H*_O_	*H*_E_	*F*_IS_	*A*_R_	*H*_O_	*H*_E_	*F*_IS_	
901	−34.133, 172.074	30	1.98	0.34	0.34	0.0003	1.98	0.34	0.36	0.0729	174.2 (46.2–∞)
902	−34.404, 172.782	30	1.98	0.36	0.35	−0.0368	1.99	0.37	0.37	0.0153	2122.7 (419.5–∞)
904	−35.695, 174.564	30	1.98	0.36	0.35	−0.0282	1.97	0.33	0.35	0.0335	1130.0 (700.0–∞)
907	−37.112, 176.868	30	1.98	0.35	0.35	−0.0188	1.95	0.32	0.34	0.0472	∞ (2886.6–∞)
908	−37.605, 177.922	14	1.97	0.36	0.34	−0.0462	1.93	0.32	0.32	0.0073	∞ (2895.2–∞)
910	−38.651, 178.169	14	1.96	0.35	0.34	−0.0343	1.93	0.31	0.33	0.0543	3951.6 (675.9–∞)
911	−39.272, 178.773	27	1.99	0.37	0.35	−0.0411	1.96	0.34	0.34	0.0113	703.3 (554.1–∞)
913	−41.211, 177.465	29	1.99	0.36	0.35	−0.0416	1.96	0.33	0.34	0.0106	1669.7 (1330.5–∞)
915	−41.476, 174.806	16	1.97	0.34	0.34	−0.0150	1.95	0.33	0.34	0.0270	∞ (409.2–∞)
916	−41.722, 174.282	30	1.98	0.34	0.35	0.0052	1.95	0.31	0.33	0.0522	8971.0 (∞–∞)
917	−42.437, 173.701	28	1.98	0.37	0.35	−0.0460	1.96	0.34	0.34	−0.0079	1345.6 (408.2–1727.9)
920	−45.551, 170.753	26	1.98	0.35	0.35	−0.0140	1.96	0.32	0.34	0.0468	1499.9 (838.5–13,000.1)
921	−45.893, 170.687	30	1.98	0.35	0.34	−0.0091	1.96	0.31	0.34	0.0702	19,941.0 (∞–∞)
924	−47.296, 167.593	29	1.98	0.34	0.34	0.0058	1.97	0.32	0.34	0.0758	153.8 (42.6–∞)
926	−46.000, 166.593	29	1.99	0.37	0.35	−0.0494	1.97	0.34	0.35	0.0030	4560.9 (387.7–∞)
927	−45.077, 167.108	30	1.98	0.35	0.35	−0.0148	1.96	0.32	0.34	0.0591	∞ (2941.6–∞)
928	−44.583, 167.807	29	1.98	0.35	0.35	−0.0055	1.97	0.32	0.33	0.0542	∞ (3784.8–∞)
933	−41.350, 174.099	29	1.98	0.36	0.35	−0.0233	1.96	0.32	0.34	0.0623	2356.4 (236.6–∞)
934	−41.111, 174.794	19	1.97	0.34	0.34	0.0110	1.94	0.31	0.33	0.0499	∞ (∞–∞)
935	−39.277, 173.722	28	1.98	0.36	0.35	−0.0268	1.99	0.35	0.36	0.0236	6757.8 (562.9–∞)
939	−35.702, 173.462	30	1.98	0.35	0.35	−0.0220	1.98	0.34	0.36	0.0461	425.4 (108.3–∞)
942	−44.349, 179.208	29	1.98	0.35	0.35	0.0007	1.96	0.32	0.34	0.0647	∞ (3034.0–∞)
TAS	−43.658, 147.403	29	1.95	0.32	0.33	0.0263	1.88	0.26	0.28	0.0943	2675.6 (207.0–∞)

Abbreviations: *A*_R_, allelic richness; *F*_IS_, inbreeding coefficient; *H*_E_, expected heterozygosity; *H*_O_, observed heterozygosity; *N*, number of individuals; *N*_e_, estimated population size under 0.001 *P*_crit_ level (95% CI based on jackknife sampling).

### 2.2 Library Preparation and Sequencing

All libraries were prepared following a modified version of the ddRADseq protocol (Peterson et al. 2012). Library preparation started with a double digest of 250 ng of high‐quality genomic DNA with frequent (*Aci*I, recognition site—CCGC) and infrequent (*Eco*RI, recognition site—GAATTC) restriction enzymes and ligation of sequencing adapters containing in‐line barcodes. Following ligation, selection of barcoded fragments was performed using AMPure XP magnetic beads (Beckman Coulter) following DeAngelis et al. (1995); fragments > 1000 and < 200 bp were removed. Illumina TruSeq LT compatible index sequences were then attached to barcoded fragments via PCR. After post‐PCR clean‐up using AMPure XP magnetic beads DNA concentrations were quantified with a Qubit 2.0 Fluorometer (Life Technologies) and standardised. Libraries were pooled with each individual contributing 25 ng of DNA: pooled library size selection was performed via an agarose gel cut with fragments between 400 and 500 bp being targeted. DNA from the excised gels was extracted using a Wizard SV Gel and PCR Clean‐Up System (Promega). Final library concentrations were quantified with a Qubit 2.0 fluorometer and concentrated, if required, using an Eppendorf Concentrator 5301. Size selection and concentration of libraries were confirmed using an Agilent ScreenTape assay and qPCR. A total of nine adult lobster libraries were sequenced on the Illumina HiSeq2500 platform with single end 100 bp HT chemistry kits at the Australian Genome Research Facility (AGRF) in Melbourne.

### 2.3 Bioinformatic Analysis—Preliminary Processing of the ddRADseq Data

The quality of sequences was assessed using the FastQC v.0.10.1 quality control tool (Andrews 2017). Reads were then trimmed to 95 bp using TRIMMOMATIC v 0.3 (Bolger et al. 2014). Trimmed reads were filtered for possible contamination using Kraken v.3.5.0 (Wood and Salzberg 2014) and were further demultiplexed using Stacks v.1.37 module ‘process_radtags’ (Catchen et al. 2013). A reference catalogue (a list of the most frequently occurring sequences throughout the dataset) was built using a Rad‐loci pipeline. The La Trobe University Rad‐loci pipeline (https://github.com/molecularbiodiversity/rad‐loci) (Villacorta‐Rath et al. 2016) was also used for a reference catalogue compilation. This pipeline involves the clustering of sequences, which was performed using VSearch v.1.1.3 (Rognes et al. 2016), with the minimal occurrence of a unique sequence set equal to the number of individuals sequenced. This promotes the selection of loci with lower levels of missing data. To merge similar reads that may have occurred from sequencing errors, the clustering identity was set to 94%, meaning a maximum 7 bp mismatch was permitted for 95 bp fragments. The clusters obtained were filtered based on the number of members in the cluster: clusters with only one member were removed as they were considered uninformative, and clusters with more than 16 members were also removed to avoid multi‐mapping. In the next step, sequences were aligned onto the prepared catalogue to estimate the number of alleles for each cluster and the catalogue was re‐filtered to retain clusters with 2 to 16 alleles. Individual sequences were mapped onto the re‐filtered catalogue once again, and loci with more than 40% missing data per locus were removed. Here ‘locus’ is used to describe a unique reference sequence 95 bp long between the *Eco*RI and *Aci*I cut sites with a cluster of mappable sequence reads.

### 2.4 SNP Calling and Characterisation

The Rad‐loci pipeline created a catalogue of reference loci and mapping of the individual reads onto the catalogue and SNP calling relied on third‐party software. Quality and contamination filtered individual reads were mapped onto the reference catalogue using the Burrows‐Wheeler Aligner v 0.7.13 (Li and Durbin 2010). Reference catalogue loci with a minimum coverage of 10× were used to minimise the effect of allele dropout (ADO). SNP calling was performed by two independent programmes: Genome Analysis Toolkit (GATK) v 3.5.0 (McKenna et al. 2010) and VarScan v 2.4.1 (Koboldt et al. 2009). Only SNPs consistently called by both programmes were selected using SelectVariants module of GATK software. SNPs in concordance identified by GATK and VarScan were further filtered using VCFtools v 0.1.14 (Danecek et al. 2011) and various R packages.

Non‐biallelic loci were removed with the *‐‐min‐alleles* 2 *‐‐max‐alleles* 2 options and only one SNP per locus was retained using the *‐‐thin* 95 option. To limit the effect of under‐represented and over‐represented loci, SNPs with a depth less than the mean of 10 (−min‐meanDP) or more than 100 (−max‐meanDP) were removed. Data missingness was set to a threshold of 20%, and rare alleles were removed using the minor allele frequency filtering (MAF) cutoff of 0.05 using the R package *poppr* v2.9.4 (Kamvar et al. 2015). The gl.filter.hwe() function of *dartR* was used to filter out loci showing significant departure from Hardy–Weinberg equilibrium (HWE) based on observed frequencies of reference homozygotes, heterozygotes and alternate homozygotes. Loci with excess observed heterozygosity (H_*O*_ > 0.6), deviating from HWE, and under linkage disequilibrium (LD; *r* ^2^ > 0.8), were also removed from the dataset using *dartR* v2.7.2 (Mijangos et al. 2022).

Markers under selection were identified using two *F*_ST_‐based approaches (BayeScan and OutFLANK) and a principal component analysis (PCA)‐based method using PCAdapt. BayeScan follow the multinomial–Dirichlet distribution model to estimate locus‐specific *F*_ST_ values and indicate the degree of differentiation between populations (Foll and Gaggiotti 2008). Following the default parameters, outlier detection using BayeScan v2.1 was performed using 20 pilot runs with 10,000 iterations and a burn‐in of 200,000 steps. Outlier SNPs were then identified using a false discovery rate (FDR) *q*‐value threshold of 0.05. OutFLANK detects outliers based on an inferred distribution of neutral *F*_ST_ values based on a maximum‐likelihood approach (Whitlock and Lotterhos 2015). OutFLANK v0.2 was run using recommended values with left and right trimming fractions set at 0.05, minimum heterozygosity of 0.1, and an FDR *q*‐value threshold of 0.05. PCAdapt detects loci under selection by identifying axes of variation that are most strongly associated with population differentiation, without requiring prior knowledge of the demographic history of the populations (Luu et al. 2017; Privé et al. 2020). PCAdapt v4.3.3 was performed by calculating statistics and *p*‐values based on the correlations between SNPs and the first *K* principal components. SNPs were then identified as outlier loci when their FDR‐adjusted *q*‐value was < 0.001. Only SNPs identified by all three outlier tests were considered as putative outliers. All loci flagged as a putative outlier by any method were excluded to generate a conservative dataset of neutral markers.

### 2.5 Genetic Diversity, Population Structure and Connectivity

Population genetics analyses were performed separately using the putatively neutral and outlier datasets. Population genetic diversity indices such as number of alleles (*N*_A_), proportion of observed alleles (*A*_O_), allelic richness (*A*_R_), observed heterozygosity (*H*_O_), expected heterozygosity (*H*_E_) and the inbreeding coefficient (*F*_IS_) were calculated using the R package *diversity* (Keenan et al. 2013). Effective population size (*N*_e_) for each sampling site was estimated from linkage disequilibrium using NeEstimator v2 (Do et al. 2014). Genetic differentiation values over all populations (global *F*_ST_) and between populations (pairwise *F*_ST_) were estimated using Weir and Cockerham (1984) *F*‐statistics in the R packages *hierfstat* v0.05‐11 (Goudet and Jombart 2022) and *dartR* v2.7.2 (Gruber et al. 2018; Mijangos et al. 2022). *F*_ST_ *p*‐values were tested for statistical significance (deviation from zero) across loci with 10,000 bootstrap replicates and results were adjusted for multiple testing using the approach of Benjamini and Hochberg (1995). Analysis of molecular variance (AMOVA; Excoffier et al. 1992) was performed to test between‐group variation (*F*_CT_) inferred from *F*_ST_ analysis. AMOVA was performed using poppr v2.8.3 (Kamvar et al. 2014), with significance testing using 1000 permutations. AMOVA results were verified using spatial analysis of molecular variance (SAMOVA) (Dupanloup et al. 2002; Excoffier and Lischer 2010), an approach to define groups of populations without a priori knowledge of sampling sites. To test for isolation‐by‐distance, correlations were estimated between Euclidean matrices of oceanographic distances (km) and genetic distances (*F*_ST_) amongst all sites. Oceanographic distances were measured using the shortest ocean path between sites using QGIS v3.34.0‐Prizren.

Population genetic structure was inferred using three approaches—Discriminant Analysis of Principal Components (DAPC; Jombart et al. 2010), STRUCTURE (Pritchard et al. 2000) and GENELAND (Guillot et al. 2005). DAPC is a multivariate analysis that uses principal component analysis (PCA) to reduce the dimensionality of genetic data and then uses discriminant analysis (DA) to assign individuals to populations based on their genetic similarity/differentiation. DAPC was performed using the R package *adegenet* v2.1.10 (Jombart 2008; Jombart and Ahmed 2011), and the number of principal components (PCs) was defined as *K*−1 (where *K* = the number of presumed populations and is equal to the number of retained PCs), which follows the recommendation of Thia (2023) in obtaining biologically meaningful results while avoiding issues of overfitting. STRUCTURE and GENELAND both use a Bayesian‐based clustering algorithm that assigns individuals to populations based on genetic signature. Both programmes follow a Markov Chain Monte Carlo (MCMC) approach to infer the number of populations (*K*) and the proportion of each individual's genome that originates from each population. STRUCTURE does not consider the geospatial relationships amongst collecting sites whereas GENELAND does. STRUCTURE v2.3.4 was run using default parameters, with burn‐in period and MCMC reps set to 5000. GENELAND v4.0.8 was performed following the recommended parameters. Initial MCMC outputs were processed to generate the final estimate of *K*, which was used as the maximum number of populations in succeeding runs (*n* = 10). The run with the highest average posterior probability was used to calculate individual membership coefficients and posterior probability of membership.

For the neutral SNPs dataset only, the migration rates amongst sampling sites were estimated. The programme *divMigrate* from the R package *diveRsity* v.1.9.90 (Keenan et al. 2013) was used to calculate the mean relative (asymmetrical) migration rates between pairs of populations across three metrics (Jost's *D*, *G*_ST_ and *N*_m_) using 1000 bootstrap iterations at a 95% confidence interval. *Migrate‐n* (Migrate‐n v4.4.4, Beerli et al. 2019) was employed to calculate mutation‐scaled migration rates (*M = m/μ*, where *m* = immigration rate per generation and *μ* = mutation rate per site) and assess the probabilities of different migration models. Model convergence was assessed from the posterior distribution plots generated by Migrate‐n, where convergence is represented in the histograms by a single distinct peak and approximate Gaussian (normal) distribution (Beerli et al. 2019). Migration models were based on patterns of genetic structure and visually compared to modelled larval connectivity of *J. edwardsii* (Chiswell and Booth 2008). Specifically, three migration models were simulated: (a) bidirectional gene flow amongst populations; (b) unidirectional gene flow from South to Northwest to Northeast (S → NW → NE); and (c) unidirectional gene flow from Northeast to Northwest to South (NE → NW → S). Migration models were executed using the Message Passage Interface (MPI) scheme with default parameters, employing Metropolis sampling and a static heating scheme featuring four chains. The simulation involved 1 × 10^6^ generations with 10 replicate chains, with sampling conducted every 100 steps, and a burn‐in period of 100,000 steps. To determine the most likely migration model, log Bayes factors (LBFs) were computed from the Bezier approximation score and used to rank all three gene flow models (Beerli and Palczewski 2010).

### 2.6 Seascape Genetics

To determine the effect of environmental factors on observed genetic differentiation, we examined correlations between site‐specific geospatial and environmental variables and site‐specific *F*_ST_ estimates (e.g., Wei et al. 2013b; Silva and Gardner 2016; Zeng et al. 2020; Holland et al. 2022). Geospatial variables included latitude, longitude and an index of Euclidean distance (km) amongst sites (e.g., Wei et al. 2013b) but preliminary analyses (data not shown) indicated that these geospatial variables were strongly correlated with variation in one or more of the environmental variables and so subsequent analyses focussed only on the environmental variables.

#### 2.6.1 Environmental Data and Variable Selection

Site‐specific environmental variables were obtained for the New Zealand sampling sites from multiple different datasets (e.g., Zeng et al. 2020; Holland et al. 2022). A total of 34 environmental variables were extracted for each sampling site (Table S1), and were tested for multicollinearity using the R packages *stats* v4.2.2 (R Core Team 2022) and *vegan* v.2.6‐4 (Oksanen et al. 2022). Variables with strong pairwise correlations (*r* ≥ 0.8) were identified, and one from each correlated pair was removed, and tested using the variance inflation factor (VIF) approach. Variable reduction was guided by ecological and/or biological knowledge of *J. edwardsii* , but in general, any environmental variable with a VIF value > 10.0 was removed to leave a dataset comprised of 16 independent environmental variables (Table S2). When any two variables are correlated and one is dropped the retained variable may be interpreted as explaining variation in the dropped variable.

#### 2.6.2 Genotype‐Environment Association Analyses

Following Wei et al. (2013b), GLMs were used to analyse the relationship between environmental variation and site‐specific mean *F*_ST_ values (the GLZ routine in STATISTICA v7.0). GLM analyses were performed using the Akaike information criterion (AIC) to rank all possible models. A test of all effects was performed using the Wald *χ* ^2^ statistic. Because of the large number of variables (*n* = 16) the dataset was split into two (*n* = 8 variables per dataset based on alphabetical order, which is effectively random) to facilitate GLM analysis. Results from the testing of the two datasets were then combined.

Following Wei et al. (2013b), Biological Environmental Stepwise (BEST) analysis was carried out using PRIMER v6 software with PERMANOVA+ (Clarke and Gorley 2006; Anderson et al. 2008). A Euclidean resemblance matrix was calculated for normalised environmental data and a Bray‐Curtis resemblance matrix was calculated for the genetic data (major allele frequencies per locus for the neutral and outlier panels separately). To test for a correlation between the two matrices, the BIOENV procedure of the BEST routine was implemented. The BIOENV procedure (Clarke 1993) aims to identify a subset of the explanatory variables whose Euclidean distance matrix correlates maximally with the Bray–Curtis dissimilarity matrix of genetic data. The procedure implements the Spearman correlation coefficient method to rank all combinations of variables. Models were considered significant at *p* < 0.05 after 999 permutations.

Following Silva and Gardner (2016), redundancy analysis (RDA), which is an extension of multiple linear regression, was employed to assess the relationship between multiple response variables and multiple explanatory variables. This method employs constrained ordination of linear combinations of explanatory variables along RDA axes that are designed to maximise their relationship with these variables. A model with the highest explanatory potential is selected according to the highest adjusted coefficient of determination. The distance‐based linear model (DISTLM) and distance‐based redundancy analysis (dbRDA) procedures (Legendre and Anderson 1999) of the PRIMER with PERMANOVA+ software package were employed to perform an ordination of fitted values from a given model and to visualise the fitted model in multidimensional space (Anderson et al. 2008).

In summary, three different seascape genetics analyses were employed to test both the neutral and the outlier loci datasets for genotype‐environment associations. The GLM is a simple linear model and tests for variation in a single dependent variable (*F*_ST_), which is itself an index of site‐specific genetic differentiation. The BEST analysis employs the raw genetic dataset (the major allele frequency for each locus) and is a rank (Spearman's coefficient) analysis, whereas the DISTLM and dbRDA also use the raw genetic dataset but attempt to fit a linear model of association between the genetic and the environmental datasets.

## 3 Results

### 3.1 Genotyping Results

During the initial filtering steps, an average of 2,969,221 sequence reads obtained from 615 adult lobsters was processed from 9 ddRADseq libraries. Average coverage was 31.91 fold and genotyping error was < 5%. A small fraction of reads (6.4%) was removed due to low‐quality scores, uncalled bases and sequences with adapter contamination. Subsequently, a reference catalogue comprising 23,169 loci was utilised, and after mapping and additional filtering 9651 loci were retained for further analysis. Amongst these loci, only 2336 SNPs were consistently identified using both GATK and VarScan tools. Following successive filtering steps (Table 2), a final filtered SNP dataset consisting of 1858 loci was obtained. BayeScan identified 139 outlier loci putatively under diversifying selection (*F*_ST_ = 0.0130 to 0.1481). OUTflank identified 125 outlier SNPs (*F*_ST_ = 0.0416 to 0.3494), of which 115 loci were also found by BayeScan. Furthermore, pcadapt identified 201 loci putatively under selection, of which 100 SNPs were identified by both BayeScan and outFLANK. Overall, 250 unique outlier loci were identified by the three outlier tests, and these were designated as the outlier loci dataset. Consequently, a SNP panel consisting of 1608 neutral loci was assigned as the neutral SNP dataset.

**TABLE 2:** Bioinformatic processes and specific filtering parameters used to generate the final SNP panels of loci for *Jasus edwardsii* .

Sequence filtering	Average number of reads per individual	2,969,221
	Average percentage of contamination‐filtered reads	3.9
	Average percentage of low quality and ambiguous barcode reads	2.5
Reference catalogue filtering	Total loci	23,169
	Filtered loci	9651
SNP filtering	Called with VarScan	159,139
	VarScan filtered	3325
	Called with GATK	211,553
	GATK filtered	7597
	VarScan/GATK concordance	2336
	No. of retained SNPs after additional filtering steps:	
	‐‐min‐meanDP10, ‐‐max‐meanDP	2211
	MAF (< 0.05)	2185
	Excess heterozygosity (> 0.06)	1859
	HWE	1859
	Linkage disequilibrium	1858
Final datasets	Neutral	1608
	Outlier	250

### 3.2 Population Genetic Analyses

The neutral loci (1608 SNPs) and the outlier loci (250 SNPs) datasets exhibited good agreement between observed and expected heterozygosity values (Table 1). Estimates of effective population size based on neutral loci only indicated large population sizes across all sites (*N*e > 400), except for New Zealand (NZ) sites 901 and 924, which displayed *N*_e_ values < 200. Both neutral and outlier loci revealed low mean inbreeding coefficients, with higher *F*_IS_ values calculated for the Australian site TAS in comparison to all NZ sites (Table 1). Additionally, TAS showed statistically significantly lower allelic richness than the NZ sites for both the neutral and outlier data sets (*t*‐tests, *p* < 0.0001 in both cases).

Analysis of global *F*_ST_ values revealed significant genetic differentiation for both the neutral (*F*_ST_ = 0.0043; *p* < 0.001) and outlier (*F*_ST_ = 0.0584; *p* < 0.001) datasets. Removing TAS revealed for the NZ‐only dataset lower genetic variation for both neutral (*F*_ST_ = 0.0017; *p* < 0.001) and outlier loci (*F*_ST_ = 0.0288; *p* < 0.001). Pairwise population differentiation using neutral markers revealed TAS as the most genetically divergent sample (*F*_ST_ = 0.0203–0.0252; significant for all site comparisons), followed by site 902 (*F*_ST_ = 0.0006–0.0206; significant in 15 of 22 pairwise comparisons) (Table 3). Significant differentiation was detected amongst the three groups, TAS, site 902, and the rest of NZ (*F*_CT_ = 0.0119, *p* < 0.001), accounting for 1.19% of the total variance. However, greater variation was calculated when tested only in two genetic groups (TAS and NZ), which corresponded to 2.20% of total genetic variance (*F*_CT_ = 0.0220, *p* < 0.001). Based on the NZ sites only, separating site 902 from all other sites revealed low but significant between‐group variation (*F*_CT_ = 0.0013; *p* > 0.001). This result was supported by SAMOVA, which suggested *K* = 2, separating site 902 from all other NZ sites (*F*_CT_ = 0.0018; *p* = 0.041).

**TABLE 3:** Pairwise population *F*_ST_ values of *Jasus edwardsii* using neutral (1608 SNPs ‐ below diagonal) and outlier loci (250 SNPs ‐ above diagonal) with *p*‐values in brackets.

Site	Northwest (NW)				Northeast (NE)												South (S)						AUS
	901	902	939	935	933	934	942	904	907	908	910	911	913	915	916	917	920	921	924	926	927	928	TAS
901		0.0006	0.0004	0.0015	0.0014	0.0007	0.0012	0.0015	0.0018	0.0011	0.0000	0.0008	0.0001	0.0000	0.0007	0.0010	0.0020	0.0025	0.0018	0.0022	0.0018	0.0007	0.0212
902	0.2479		0.0006	0.0012	0.0018	0.0021	0.0014	0.0022	0.0025	0.0017	0.0025	0.0024	0.0017	0.0025	0.0017	0.0025	0.0032	0.0030	0.0027	0.0014	0.0018	0.0009	0.0206
939	0.3692	0.2591		0.0010	0.0006	0.0009	0.0002	0.0017	0.0000	0.0020	0.0000	0.0000	0.0005	0.0005	0.0005	0.0009	0.0012	0.0014	0.0025	0.0016	0.0014	0.0002	0.0203
935	*0.0458*	0.0681	0.1569		0.0004	0.0006	0.0000	0.0009	0.0009	0.0018	0.0009	0.0004	0.0008	0.0011	0.0000	0.0018	0.0013	0.0012	0.0003	0.0020	0.0003	0.0008	0.0205
933	0.0597	*0.0167*	0.2591	0.3692		0.0001	0.0000	0.0007	0.0006	0.0014	0.0011	0.0002	0.0009	0.0005	0.0003	0.0014	0.0009	0.0018	0.0025	0.0016	0.0013	0.0014	0.0236
934	0.2905	*0.0304*	0.2301	0.3101	0.5514		0.0006	0.0004	0.0000	0.0011	0.0000	0.0013	0.0004	0.0000	0.0008	0.0019	0.0020	0.0009	0.0013	0.0023	0.0020	0.0000	0.0224
942	0.0933	0.0706	0.4507	0.6454	0.7269	0.3661		0.0005	0.0003	0.0001	0.0000	0.0003	0.0000	0.0003	0.0000	0.0000	0.0005	0.0011	0.0008	0.0007	0.0006	0.0002	0.0235
904	*0.0418*	*0.0030*	*0.0190*	0.1764	0.1903	0.4251	0.3252		0.0009	0.0017	0.0000	0.0010	0.0013	0.0015	0.0000	0.0016	0.0004	0.0011	0.0009	0.0004	0.0006	0.0000	0.0214
907	*0.0231*	*0.0000*	0.6864	0.1864	0.2591	0.5921	0.4383	0.1846		0.0019	0.0006	0.0008	0.0016	0.0013	0.0000	0.0012	0.0018	0.0003	0.0018	0.0022	0.0013	0.0005	0.0242
908	0.2522	0.0814	0.0650	0.0976	0.1718	0.3061	0.5445	0.1055	0.0988		0.0021	0.0030	0.0006	0.0017	0.0005	0.0011	0.0017	0.0011	0.0021	0.0031	0.0023	0.0000	0.0238
910	0.6887	*0.0231*	0.7597	0.2896	0.2166	0.6094	0.6944	0.6207	0.3639	0.1657		0.0002	0.0007	0.0008	0.0000	0.0009	0.0010	0.0011	0.0001	0.0003	0.0000	0.0007	0.0240
911	0.1928	*0.0016*	0.5546	0.3641	0.4507	0.1645	0.4279	0.1569	0.1896	*0.0190*	0.5245		0.0000	0.0000	0.0008	0.0012	0.0014	0.0016	0.0027	0.0018	0.0006	0.0000	0.0223
913	0.5153	*0.0299*	0.2896	0.1864	0.1625	0.4048	0.6482	0.0626	*0.0392*	0.3917	0.3396	0.6860		0.0012	0.0009	0.0016	0.0024	0.0018	0.0017	0.0012	0.0008	0.0005	0.0222
915	0.6002	*0.0190*	0.3832	0.1864	0.3704	0.8349	0.4708	0.1150	0.1696	0.1903	0.3661	0.8349	0.1696		0.0000	0.0017	0.0027	0.0013	0.0019	0.0015	0.0015	0.0012	0.0234
916	0.2073	*0.0231*	0.2896	0.8928	0.3930	0.2579	0.6207	0.6944	0.5719	0.4073	0.5603	0.2174	0.1718	0.6887		0.0012	0.0011	0.0008	0.0003	0.0018	0.0000	0.0000	0.0233
917	0.1348	*0.0009*	0.1696	*0.0205*	*0.0419*	*0.0392*	0.5618	*0.0332*	0.0912	0.2066	0.2705	0.0971	*0.0302*	0.0933	0.0814		0.0018	0.0006	0.0018	0.0014	0.0016	0.0012	0.0240
920	*0.0114*	*0.0000*	0.0932	0.0814	0.1804	0.0626	0.3101	0.3692	*0.0357*	0.1162	0.2474	0.0756	*0.0023*	*0.0220*	0.1203	*0.0190*		0.0018	0.0016	0.0010	0.0000	0.0000	0.0252
921	*0.0023*	*0.0000*	0.0756	0.0844	*0.0202*	0.2301	0.1112	0.1071	0.4014	0.2228	0.2328	*0.0453*	*0.0175*	0.1784	0.1903	0.2905	*0.0304*		0.0008	0.0009	0.0005	0.0000	0.0245
924	*0.0203*	*0.0009*	*0.0016*	0.4251	*0.0016*	0.1645	0.1985	0.1645	*0.0204*	0.0654	0.5454	*0.0009*	*0.0296*	0.0706	0.3832	*0.0231*	*0.0458*	0.2142		0.0006	0.0011	0.0004	0.0243
926	*0.0062*	0.0626	*0.0330*	*0.0043*	*0.0299*	*0.0190*	0.2312	0.3101	*0.0062*	*0.0067*	0.4316	*0.0315*	0.0932	0.1112	*0.0196*	*0.0500*	0.1645	0.1645	0.2474		0.0008	0.0002	0.0247
927	*0.0190*	*0.0114*	*0.0453*	0.4251	0.0631	*0.0453*	0.2522	0.2591	0.0813	*0.0458*	0.7269	0.2755	0.2049	0.1497	0.6103	*0.0392*	0.5514	0.3057	0.1307	0.1764		0.0000	0.0227
928	0.2307	0.1479	0.4837	0.1696	0.0691	0.5921	0.4911	0.6094	0.2905	0.6864	0.3224	0.5528	0.3330	0.1903	0.6090	0.1112	0.8106	0.6207	0.3763	0.4279	0.8918		0.0242
TAS	*0.0000*	*0.0000*	*0.0000*	*0.0000*	*0.0000*	*0.0000*	*0.0000*	*0.0000*	*0.0000*	*0.0000*	*0.0000*	*0.0000*	*0.0000*	*0.0000*	*0.0000*	*0.0000*	*0.0000*	*0.0000*	*0.0000*	*0.0000*	*0.0000*	*0.0000*	
901		0.0058 (0.0053)	0.0072 (0.0042)	0.0095 (0.0000)	0.0340 (0.0000)	0.0304 (0.0000)	0.0348 (0.0000)	0.0279 (0.0000)	0.0345 (0.0000)	0.0384 (0.0000)	0.0346 (0.0000)	0.0334 (0.0000)	0.0323 (0.0000)	0.0259 (0.0000)	0.0352 (0.0000)	0.0353 (0.0000)	0.0297 (0.0000)	0.0248 (0.0000)	0.0215 (0.0000)	0.0212 (0.0000)	0.0234 (0.0000)	0.0267 (0.0000)	0.2746 (0.0000)
902	0.0006 (0.2479)		0.0099 (0.0000)	0.0101 (0.0001)	0.0269 (0.0000)	0.0310 (0.0000)	0.0323 (0.0000)	0.0235 (0.0000)	0.0299 (0.0000)	0.0321 (0.0000)	0.0320 (0.0000)	0.0313 (0.0000)	0.0246 (0.0000)	0.0238 (0.0000)	0.0298 (0.0000)	0.0287 (0.0000)	0.0290 (0.0000)	0.0260 (0.0000)	0.0294 (0.0000)	0.0225 (0.0000)	0.0252 (0.0000)	0.0327 (0.0000)	0.2583 (0.0000)
939	0.0004 (0.3692)	0.0006 (0.2591)		0.0031 (0.0516)	0.0345 (0.0000)	0.0412 (0.0000)	0.0462 (0.0000)	0.0259 (0.0000)	0.0349 (0.0000)	0.0424 (0.0000)	0.0399 (0.0000)	0.0342 (0.0000)	0.0379 (0.0000)	0.0336 (0.0000)	0.0320 (0.0000)	0.0424 (0.0000)	0.0226 (0.0000)	0.0186 (0.0000)	0.0220 (0.0000)	0.0226 (0.0000)	0.0211 (0.0000)	0.0201 (0.0000)	0.2779 (0.0000)
935	0.0015 (0.0458)	0.0012 (0.0681)	0.0010 (0.1569)		0.0202 (0.0000)	0.0266 (0.0000)	0.0265 (0.0000)	0.0164 (0.0000)	0.0197 (0.0000)	0.0277 (0.0000)	0.0208 (0.0000)	0.0188 (0.0000)	0.0241 (0.0000)	0.0189 (0.0000)	0.0180 (0.0000)	0.0293 (0.0000)	0.0107 (0.0000)	0.0072 (0.0001)	0.0126 (0.0000)	0.0122 (0.0000)	0.0075 (0.0007)	0.0100 (0.0001)	0.2809 (0.0000)
933	0.0014 (0.0597)	0.0018 (0.0167)	0.0006 (0.2591)	0.0004 (0.3692)		0.0035 (0.1236)	0.0041 (0.0426)	0.0009 (0.3246)	0.0029 (0.0565)	0.0007 (0.4217)	0.0003 (0.4827)	0.0012 (0.2787)	0.0081 (0.0024)	0.0000 (0.6127)	0.0000 (0.9046)	0.0024 (0.2225)	0.0236 (0.0000)	0.0220 (0.0000)	0.0391 (0.0000)	0.0352 (0.0000)	0.0313 (0.0000)	0.0438 (0.0000)	0.3212 (0.0000)
934	0.0007 (0.2905)	0.0021 (0.0304)	0.0009 (0.2301)	0.0006 (0.3101)	0.0001 (0.5514)		0.0010 (0.3484)	0.0103 (0.0007)	0.0067 (0.0252)	0.0145 (0.0001)	0.0118 (0.0043)	0.0087 (0.0052)	0.0076 (0.0051)	0.0049 (0.0830)	0.0066 (0.0411)	0.0067 (0.0065)	0.0296 (0.0000)	0.0302 (0.0000)	0.0360 (0.0000)	0.0369 (0.0000)	0.0378 (0.0000)	0.0486 (0.0000)	0.3345 (0.0000)
942	0.0012 (0.0933)	0.0014 (0.0706)	0.0002 (0.4507)	0.0000 (0.6454)	0.0000 (0.7269)	0.0006 (0.3661)		0.0067 (0.0004)	0.0045 (0.0183)	0.0085 (0.0062)	0.0019 (0.3160)	0.0024 (0.1277)	0.0048 (0.0259)	0.0018 (0.2594)	0.0019 (0.2413)	0.0052 (0.0241)	0.0227 (0.0000)	0.0292 (0.0000)	0.0358 (0.0000)	0.0328 (0.0000)	0.0348 (0.0000)	0.0423 (0.0000)	0.3369 (0.0000)
904	0.0015 (0.0418)	0.0022 (0.0030)	0.0017 (0.0190)	0.0009 (0.1764)	0.0007 (0.1903)	0.0004 (0.4251)	0.0005 (0.3252)		0.0034 (0.0400)	0.0080 (0.0178)	0.0044 (0.0870)	0.0000 (0.8077)	0.0094 (0.0025)	0.0062 (0.0142)	0.0029 (0.1021)	0.0096 (0.0006)	0.0158 (0.0000)	0.0194 (0.0000)	0.0341 (0.0000)	0.0312 (0.0000)	0.0276 (0.0000)	0.0348 (0.0000)	0.3108 (0.0000)
907	0.0018 (0.0231)	0.0025 (0.0000)	0.0000 (0.6864)	0.0009 (0.1864)	0.0006 (0.2591)	0.0000 (0.5921)	0.0003 (0.4383)	0.0009 (0.1846)		0.0066 (0.0352)	0.0036 (0.1548)	0.0038 (0.0255)	0.0099 (0.0001)	0.0062 (0.0146)	0.0000 (0.5705)	0.0102 (0.0001)	0.0253 (0.0000)	0.0261 (0.0000)	0.0382 (0.0000)	0.0340 (0.0000)	0.0321 (0.0000)	0.0448 (0.0000)	0.3260 (0.0000)
908	0.0011 (0.2522)	0.0017 (0.0814)	0.0020 (0.0650)	0.0018 (0.0976)	0.0014 (0.1718)	0.0011 (0.3061)	0.0001 (0.5445)	0.0017 (0.1055)	0.0019 (0.0988)		0.0093 (0.0184)	0.0075 (0.0085)	0.0064 (0.0342)	0.0100 (0.0041)	0.0000 (0.5983)	0.0116 (0.0021)	0.0332 (0.0000)	0.0356 (0.0000)	0.0410 (0.0000)	0.0430 (0.0000)	0.0477 (0.0000)	0.0533 (0.0000)	0.3383 (0.0000)
910	0.0000 (0.6887)	0.0025 (0.0231)	0.0000 (0.7597)	0.0009 (0.2896)	0.0011 (0.2166)	0.0000 (0.6094)	0.0000 (0.6944)	0.0000 (0.6207)	0.0006 (0.3639)	0.0021 (0.1657)		0.0045 (0.0870)	0.0073 (0.0261)	0.0044 (0.1433)	0.0000 (0.5708)	0.0047 (0.0968)	0.0192 (0.0000)	0.0221 (0.0000)	0.0370 (0.0000)	0.0307 (0.0000)	0.0253 (0.0000)	0.0375 (0.0000)	0.3339 (0.0000)
911	0.0008 (0.1928)	0.0024 (0.0016)	0.0000 (0.5546)	0.0004 (0.3641)	0.0002 (0.4507)	0.0013 (0.1645)	0.0003 (0.4279)	0.0010 (0.1569)	0.0008 (0.1896)	0.0030 (0.0190)	0.0002 (0.5245)		0.0098 (0.0008)	0.0041 (0.0809)	0.0000 (0.5705)	0.0104 (0.0007)	0.0166 (0.0000)	0.0212 (0.0000)	0.0348 (0.0000)	0.0308 (0.0000)	0.0319 (0.0000)	0.0348 (0.0000)	0.3306 (0.0000)
913	0.0001 (0.5153)	0.0017 (0.0299)	0.0005 (0.2896)	0.0008 (0.1864)	0.0009 (0.1625)	0.0004 (0.4048)	0.0000 (0.6482)	0.0013 (0.0626)	0.0016 (0.0392)	0.0006 (0.3917)	0.0007 (0.3396)	0.0000 (0.6860)		0.0048 (0.0537)	0.0078 (0.0065)	0.0044 (0.0231)	0.0247 (0.0000)	0.0240 (0.0000)	0.0289 (0.0000)	0.0241 (0.0000)	0.0332 (0.0000)	0.0434 (0.0000)	0.3325 (0.0000)
915	0.0000 (0.6002)	0.0025 (0.0190)	0.0005 (0.3832)	0.0011 (0.1864)	0.0005 (0.3704)	0.0000 (0.8349)	0.0003 (0.4708)	0.0015 (0.1150)	0.0013 (0.1696)	0.0017 (0.1903)	0.0008 (0.3661)	0.0000 (0.8349)	0.0012 (0.1696)		0.0050 (0.0681)	0.0029 (0.1182)	0.0240 (0.0000)	0.0248 (0.0000)	0.0328 (0.0000)	0.0327 (0.0000)	0.0315 (0.0000)	0.0432 (0.0000)	0.3251 (0.0000)
916	0.0007 (0.2073)	0.0017 (0.0231)	0.0005 (0.2896)	0.0000 (0.8928)	0.0003 (0.3930)	0.0008 (0.2579)	0.0000 (0.6207)	0.0000 (0.6944)	0.0000 (0.5719)	0.0005 (0.4073)	0.0000 (0.5603)	0.0008 (0.2174)	0.0009 (0.1718)	0.0000 (0.6887)		0.0052 (0.0415)	0.0204 (0.0000)	0.0198 (0.0000)	0.0354 (0.0000)	0.0326 (0.0000)	0.0320 (0.0000)	0.0367 (0.0000)	0.3291 (0.0000)
917	0.0010 (0.1348)	0.0025 (0.0009)	0.0009 (0.1696)	0.0018 (0.0205)	0.0014 (0.0419)	0.0019 (0.0392)	0.0000 (0.5618)	0.0016 (0.0332)	0.0012 (0.0912)	0.0011 (0.2066)	0.0009 (0.2705)	0.0012 (0.0971)	0.0016 (0.0302)	0.0017 (0.0933)	0.0012 (0.0814)		0.0280 (0.0000)	0.0281 (0.0000)	0.0410 (0.0000)	0.0312 (0.0000)	0.0375 (0.0000)	0.0490 (0.0000)	0.3333 (0.0000)
920	0.0020 (0.0114)	0.0032 (0.0000)	0.0012 (0.0932)	0.0013 (0.0814)	0.0009 (0.1804)	0.0020 (0.0626)	0.0005 (0.3101)	0.0004 (0.3692)	0.0018 (0.0357)	0.0017 (0.1162)	0.0010 (0.2474)	0.0014 (0.0756)	0.0024 (0.0023)	0.0027 (0.0220)	0.0011 (0.1203)	0.0018 (0.0190)		0.0016 (0.2209)	0.0104 (0.0003)	0.0085 (0.0026)	0.0060 (0.0037)	0.0055 (0.0107)	0.3329 (0.0000)
921	0.0025 (0.0023)	0.0030 (0.0000)	0.0014 (0.0756)	0.0012 (0.0844)	0.0018 (0.0202)	0.0009 (0.2301)	0.0011 (0.1112)	0.0011 (0.1071)	0.0003 (0.4014)	0.0011 (0.2228)	0.0011 (0.2328)	0.0016 (0.0453)	0.0018 (0.0175)	0.0013 (0.1784)	0.0008 (0.1903)	0.0006 (0.2905)	0.0018 (0.0304)		0.0097 (0.0006)	0.0067 (0.0095)	0.0057 (0.0036)	0.0028 (0.0723)	0.3279 (0.0000)
924	0.0018 (0.0203)	0.0027 (0.0009)	0.0025 (0.0016)	0.0003 (0.4251)	0.0025 (0.0016)	0.0013 (0.1645)	0.0008 (0.1985)	0.0009 (0.1645)	0.0018 (0.0204)	0.0021 (0.0654)	0.0001 (0.5454)	0.0027 (0.0009)	0.0017 (0.0296)	0.0019 (0.0706)	0.0003 (0.3832)	0.0018 (0.0231)	0.0016 (0.0458)	0.0008 (0.2142)		0.0002 (0.4936)	0.0090 (0.0011)	0.0038 (0.0760)	0.3174 (0.0000)
926	0.0022 (0.0062)	0.0014 (0.0626)	0.0016 (0.0330)	0.0020 (0.0043)	0.0016 (0.0299)	0.0023 (0.0190)	0.0007 (0.2312)	0.0004 (0.3101)	0.0022 (0.0062)	0.0031 (0.0067)	0.0003 (0.4316)	0.0018 (0.0315)	0.0012 (0.0932)	0.0015 (0.1112)	0.0018 (0.0196)	0.0014 (0.0500)	0.0010 (0.1645)	0.0009 (0.1645)	0.0006 (0.2474)		0.0061 (0.0074)	0.0054 (0.0382)	0.3223 (0.0000)
927	0.0018 (0.0190)	0.0018 (0.0114)	0.0014 (0.0453)	0.0003 (0.4251)	0.0013 (0.0631)	0.0020 (0.0453)	0.0006 (0.2522)	0.0006 (0.2591)	0.0013 (0.0813)	0.0023 (0.0458)	0.0000 (0.7269)	0.0006 (0.2755)	0.0008 (0.2049)	0.0015 (0.1497)	0.0000 (0.6103)	0.0016 (0.0392)	0.0000 (0.5514)	0.0005 (0.3057)	0.0011 (0.1307)	0.0008 (0.1764)		0.0027 (0.1038)	0.3241 (0.0000)
928	0.0007 (0.2307)	0.0009 (0.1479)	0.0002 (0.4837)	0.0008 (0.1696)	0.0014 (0.0691)	0.0000 (0.5921)	0.0002 (0.4911)	0.0000 (0.6094)	0.0005 (0.2905)	0.0000 (0.6864)	0.0007 (0.3224)	0.0000 (0.5528)	0.0005 (0.3330)	0.0012 (0.1903)	0.0000 (0.6090)	0.0012 (0.1112)	0.0000 (0.8106)	0.0000 (0.6207)	0.0004 (0.3763)	0.0002 (0.4279)	0.0000 (0.8918)		0.3285 (0.0000)
TAS	0.0212 (0.0000)	0.0206 (0.0000)	0.0203 (0.0000)	0.0205 (0.0000)	0.0236 (0.0000)	0.0224 (0.0000)	0.0235 (0.0000)	0.0214 (0.0000)	0.0242 (0.0000)	0.0238 (0.0000)	0.0240 (0.0000)	0.0223 (0.0000)	0.0222 (0.0000)	0.0234 (0.0000)	0.0233 (0.0000)	0.0240 (0.0000)	0.0252 (0.0000)	0.0245 (0.0000)	0.0243 (0.0000)	0.0247 (0.0000)	0.0227 (0.0000)	0.0242 (0.0000)	

*Note:* Pairwise *F*_ST_ values (and *p*‐values in brackets) from tests of differentiation using neutral (below diagonal) and outlier loci (above diagonal). Significant differences are highlighted (*p* ≤ 0.05). All *p*‐values were calculated using 10,000 bootstraps, adjusted for false discovery rate (FDR). Pairwise *F*_ST_ values (above diagonal) and *p*‐values from tests of differentiation (below diagonal). Significant differences are highlighted (*p* ≤ 0.05). All *p*‐values were calculated using 10,000 bootstraps, adjusted for false discovery rate (FDR).

**FIGURE 2:**
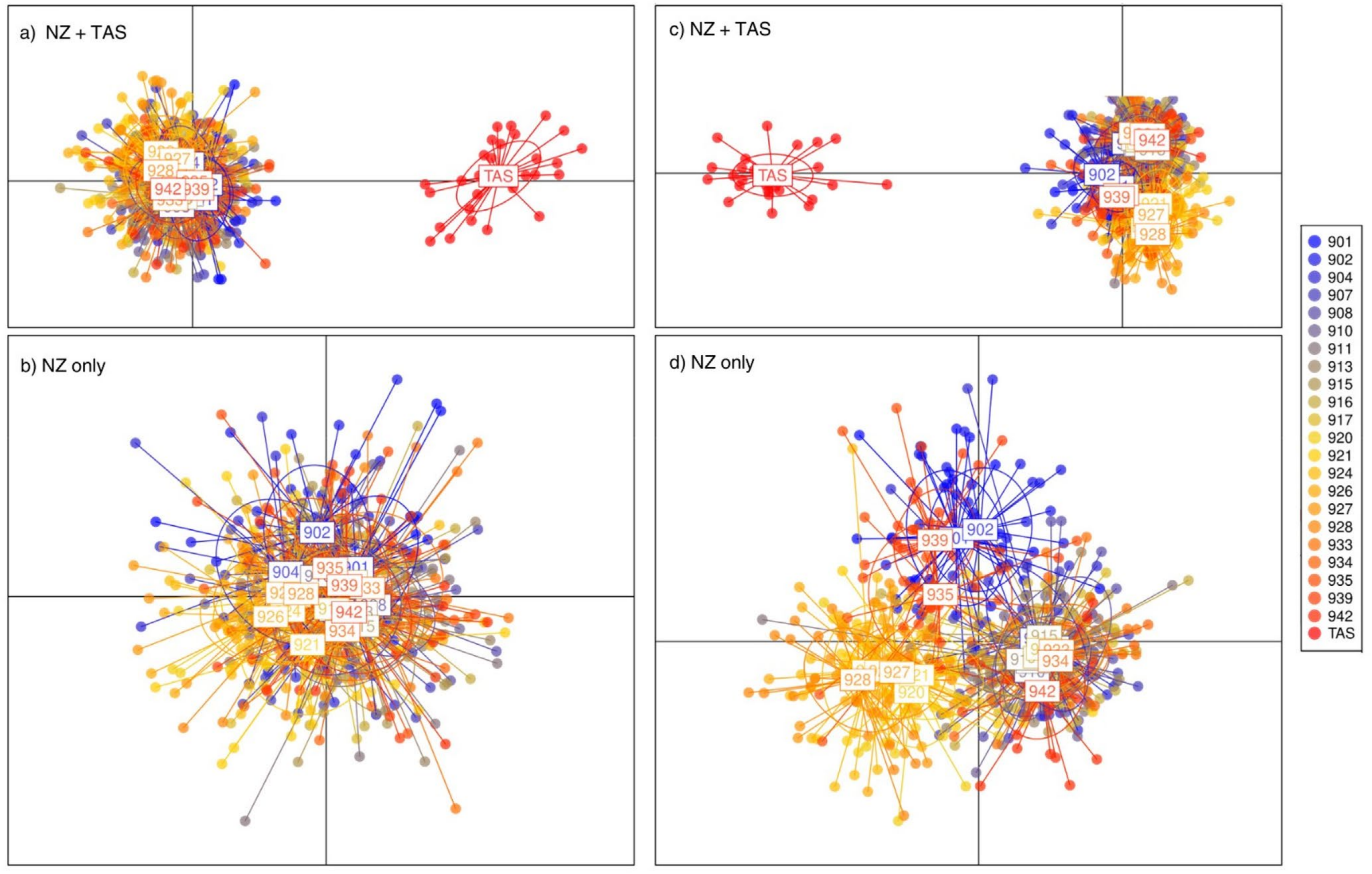
Discriminant Analysis of Principal Components (DAPC) plots of *Jasus edwardsii* individuals using neutral (a, b) and outlier datasets (c, d). Plots a and c include all individuals (NZ + TAS), while plots b and d demonstrate the spatial genetic distribution without TAS. All DAPC analyses were performed using 21 principal components. Each dot in the plot represents one individual rock lobster from a given colour‐coded sample.

**FIGURE 3:**
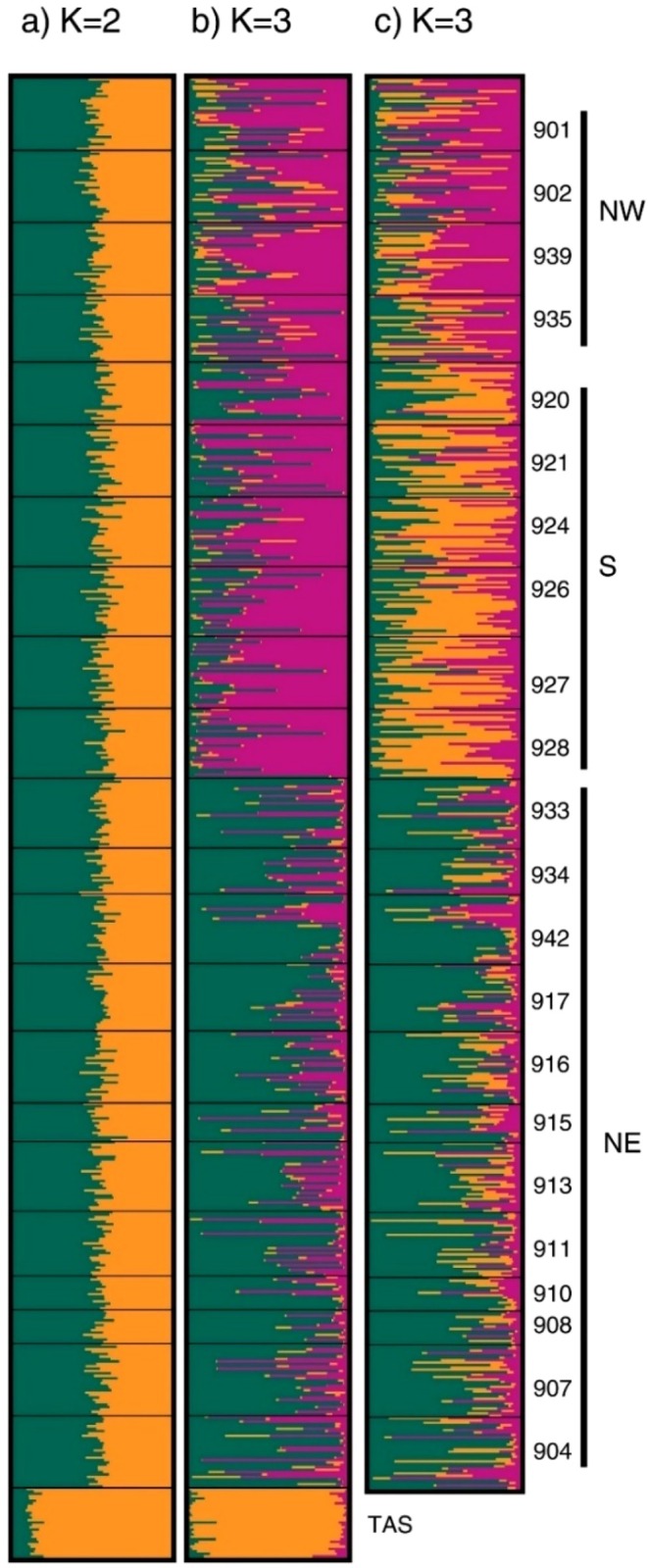
Bayesian clustering analysis of all *Jasus edwardsii* individuals using neutral (a) and outlier markers (b, c). Panel (a) shows the membership probabilities of all individuals based on neutral markers for Δ*K* = 2. Panels (b) and (c) represent clustering results based on outlier loci, with panel (b) indicating Δ*K* = 3 and panel (c) showing the genetic assignments when TAS was excluded. NZ localities were arranged based on the three genetic groups (NW, S, NE).

The DAPC analysis of neutral loci revealed significant genetic divergence between NZ sites and TAS (Figure 2a). The first two discriminant functions (DF) accounted for 80.65% and 3.72% of the variance, respectively. Subsequently, retaining only NZ sites for further analysis revealed separation of site 902 from all other sites (Figure 2b). The first two DFs explained 20.10% and 14.18% of the total variance, revealing evidence of the subtle genetic divergence of 902 from the rest of the NZ sites. STRUCTURE analysis revealed explicit genetic differentiation (Δ*K* = 2) between TAS and the NZ sites (Figure 3a). Excluding TAS from the analysis highlighted the similarity of rock lobster samples from the NZ sites. Despite the inclusion of geospatial information, GENELAND did not detect any genetic structure across NZ using the neutral markers.

Outlier locus analysis revealed significant genetic differentiation between the rock lobsters of the TAS site and the NZ sites, with TAS exhibiting the highest degree of genetic differentiation (*F*_ST_ = 0.2583–0.3383; significant for all site comparisons). Pairwise *F*_ST_ analysis also identified genetic subdivisions amongst the 22 NZ sample sites, indicating low genetic differentiation amongst sites located on the east coast of North Island (NZ sites 904, 907, 908, 910, 911, 913, 915), Chatham Islands (site 942) and the northern South Island (sites 933, 934, 916, 917), collectively referred to as the ‘NE’ region (significant in 36 of 66 pairwise comparisons; Table 3). The NE region showed pronounced differentiation from the western part of the North Island (NZ sites 935, 939, 901 and 902; hereafter referred to as the ‘NW’ region) as well as from the southernmost areas of South Island (NZ sites 920, 921, 924, 926, 927 and 928; hereafter referred to as the ‘S’ region).

AMOVA confirmed significant population differentiation amongst the identified regions: NE, NW, S and TAS, with a total between‐groups variation of 2.56% (*F*_CT_ = 0.0256; *p* < 0.001). SAMOVA further supported the presence of three distinct groups (*K* = 3) within the NZ samples (*F*_CT_ = 0.0265; *p* > 0.001). However, site 935 was assigned to the S region, not the NW region. The DAPC clearly demonstrated spatial genetic differentiation between the NZ samples and the TAS sample (Figure 2c), where the first two DFs accounted for 72.56% and 13.61% of the total variance, respectively. Upon excluding the TAS sample from the analysis, genetic differentiation was observed amongst the three NZ regions and site 935 was located between the NW and S regions (Figure 2d). The spatial genetic structure was explained by the first 2 DFs, which accounted for 20.10% and 14.18% of the variance, respectively.

**FIGURE 4:**
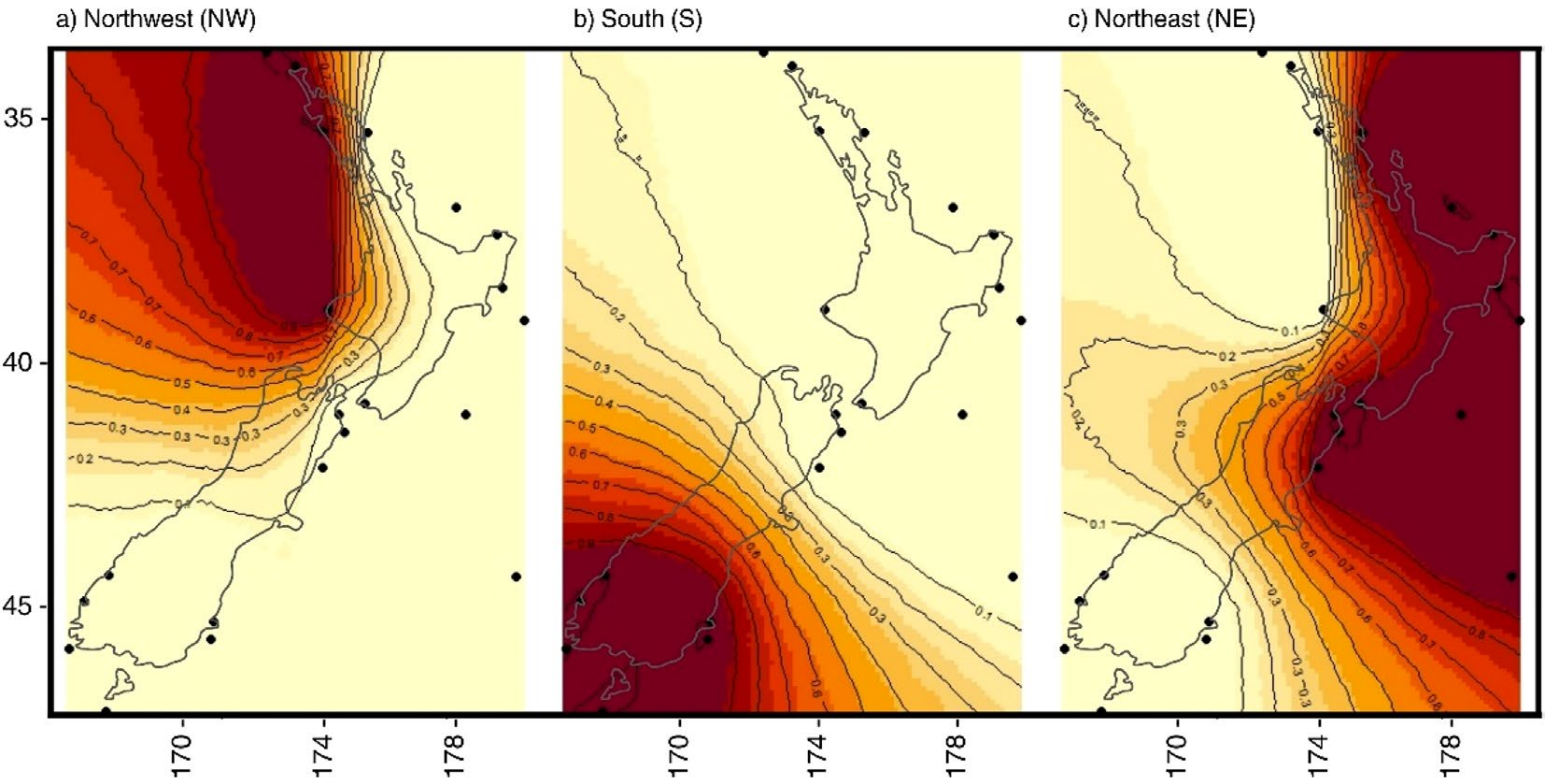
Spatial genetic structure of *Jasus edwardsii* across New Zealand based on GENELAND analysis of outlier loci. Isoclines of posterior probabilities illustrate the pronounced differentiation of genetic regions, namely (a) NE, (b) S and (c) NW. The sampling sites are represented by black dots and darker colours indicate higher probabilities of membership of each of the three identified clusters.

STRUCTURE analysis detected three distinct groups using all samples from NZ and the single sample from TAS (Δ*K* = 3; Figure 3b). STRUCTURE plots showed different genetic signatures for the (1) NW and S regions, (2) NE and (3) TAS. Removal of TAS from the analysis revealed two distinct groups in NZ (Δ*K* = 2), retaining the grouping of (1) NW‐S and (2) NE (Figure 3c). Analysis of individual ancestry proportions revealed that samples from the NW region showed subtle differentiation from samples of the S region. The detection of geospatially explicit genetic structure using GENELAND also supported the presence of three distinct groups (Figure 4), consistent with the findings from the *F*_ST_, AMOVA, DAPC and STRUCTURE (when *K* = 3) analyses.

**FIGURE 5:**
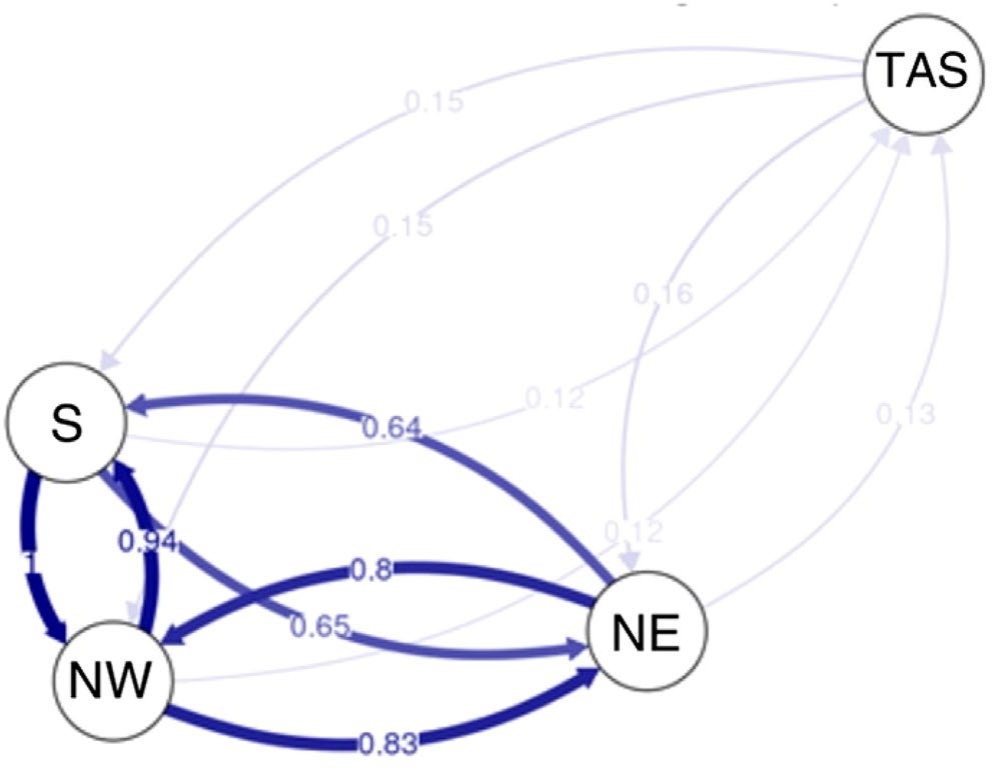
Relative migration network of pooled *Jasus edwardsii* individuals in three different regions in New Zealand (NE, NW, S) and Tasmania (TAS) calculated using Jost's *D* metric.

Relative migration estimates derived from divMigrate using the neutral dataset highlighted that the Australian rock lobsters (TAS) displayed limited bidirectional gene exchange with the NZ sites (relative values of 0.12–0.16). Within NZ, there were high levels of bidirectional gene flow amongst rock lobsters of the NW, NE and S regions (0.64–1), and an almost symmetrical pattern of gene flow between pairs of sites (Figure 5). On the other hand, while coalescent simulations using Migrate‐n (see Figure S3 for convergence plots) suggested that the model representing bidirectional gene flow amongst NZ sites had the strongest support (Table 4), asymmetric migration patterns were evident. The immigration rate (*M*) of individuals from NW to NE was higher than for NE to NW, at 56.8 (95% highest posterior density (HPD): 16.7–153.3) and 34.1 (95% HPD: 35.0–133.3), respectively. Moreover, *M* rates from S to NE were slightly higher than from NE to S, at 56.7 (95% HPD: 35.0–133.3) and 46.2 (95% HPD: 10.0–143.3), respectively. The model for unidirectional gene flow from S → NW → NE had a higher probability (greater support) than that for unidirectional gene flow from NE → NW → S.

**TABLE 4:** Log Bayes factors (LBFs) calculated from the Bezier approximation scores for three models of migration between NE, NW and S populations of *Jasus edwardsii.* LBF was calculated using the *BF* function in R package *mtraceR* (Paciani 2023).

Model	Log(mL)	LBF	Rank
Bidirectional	−112,386.15	0.00	1
S → NW → NE	−125,370.75	−25,969.20	2
NE → NW → S	−125,827.12	−26,881.94	3

### 3.3 Seascape Genetics

#### 3.3.1 Environmental Variable Selection

In total, 25 independent environmental variables were retained after pairwise correlation testing (Figure S1). This was further adjusted by calculating the variance inflation factor (VIF) for each of the 25 variables, resulting in a reduced dataset of 16 variables (Figure S2, Table S2).

#### 3.3.2 Generalised Linear Model

The pairwise *F*_*ST*_ values between sampling sites for the neutral dataset ranged from < 0.0001 to 0.0032 with calculated mean *F*_ST_ values ranging from 0.0004 for statistical area 928 to 0.0019 for area 902. For the outlier loci, these values were < 0.0001 to 0.0533 for pairwise values and 0.0152 (site 904) to 0.0296 (site 928) (Table 3). GLM analyses of the neutral dataset identified 174 of 400 models as being significant (*p* < 0.05), while the all‐effects analyses identified four environmental variables (TC, Rough, MLD, SST – *p* < 0.0001 in all cases) as being significant amongst the 16 environmental variables. For the outlier dataset, only 14 of 400 models were significant (*p* < 0.05), and only one environmental variable (Carbonate, *p* = 0.0084) was identified as explaining significant variation in mean *F*_ST_ values.

#### 3.3.3 Biological Environmental Stepwise

BEST analyses showed that the best‐fit model for the neutral dataset (*r*_s_ = 0.196, *p* = 0.058) and for the outlier dataset (*r*_s_ = 0.256, *p* = 0.390) did not explain significant variation in the genetic dataset.

#### 3.3.4 DISTLM and dbRDA

**FIGURE 6:**
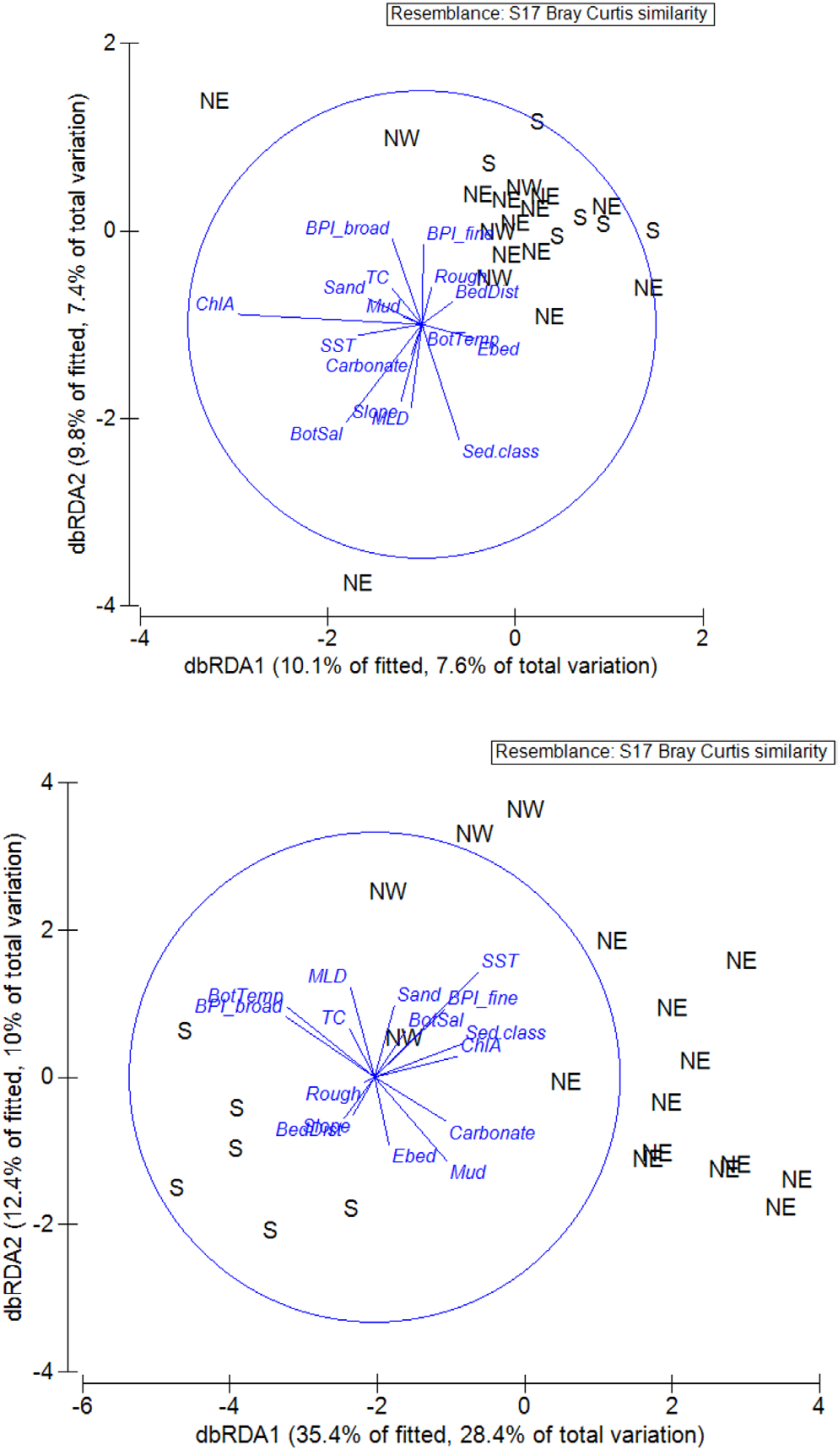
dbRDA plots from DISTLM analysis of neutral (A) and outlier loci (B) datasets of *Jasus edwardsii* . Individual sampling sites are coded according to the STRUCTURE results for *K* = 3 groups for New Zealand (NW—sites 901, 902, 935, 939; S—sites 920, 921, 924, 926, 927, 928; NE – sites 904, 907, 908, 910, 911, 913, 915, 916, 917, 933, 934, 942; refer to Figure 1 for site locations).

DISTLM analysis of the neutral dataset revealed that all marginal tests (individual effects) were not statistically significant (*p* > 0.05 in all cases). Sequential tests (inclusion of multiple environmental variables in the model) were also all not significant. The dbRDA plot revealed very little evidence of separation amongst the sampling areas (Figure 6A) although sites 908 (top left, coded as NE) and 915 (bottom, coded as NE) were both separated from the main cluster of points. In contrast, DISTLM analysis of the outlier dataset revealed that ROUGH (*p* = 0.016) and SST (*p* = 0.047) explained significant variation in the genetic dataset. The dbRDA plot (Figure 6B) revealed clear separation of the southern‐most sites (920, 921, 924, 926, 927 and 928) and the northern‐most sites (901, 902 and 939) from all other sampling sites; site 935 from the western‐most region of the North Island was an isolated single point, and all other sampling sites tended to fall loosely together.

## 4 Discussion

Understanding patterns of connectivity and adaptive divergence is increasingly important for the conservation and sustainable management of harvested species. This study aimed to assess the genetic connectivity, genetic structure and adaptive divergence of the red rock lobster, *Jasus edwardsii* , across its entire New Zealand (NZ) range, and with one reference sample from Australia (TAS ‐ Tasmania), using two types of genetic markers: neutral and outlier SNPs. The results confirmed significant genetic differentiation between the sample from Tasmania (TAS) and those from NZ using both locus types. At the NZ scale, analysis with neutral loci revealed a high degree of genetic connectivity of *J. edwardsii* , but with subtle genetic differences observed at site 902 (situated in northern NZ). This aligns with earlier oceanographic modelling work that suggests widespread larval mixing throughout NZ (Chiswell and Booth 2008, 2017). Despite this connectivity, analysis with outlier markers identified three genetically distinct groups within NZ: Northeast (NE), Northwest (NW) and Southern (S). Migration models demonstrated an asymmetric pattern of gene flow between these genetic groups, likely due to persistent oceanographic features. Genetic structure observed for outlier but not for neutral markers was strongly correlated with putatively adaptive forces amongst various environmental variables in NZ.

### 4.1 Population Genetics

#### 4.1.1 Genetic Differentiation Between New Zealand and Australia

Both neutral and outlier marker panels of single nucleotide polymorphisms (SNPs) derived from *J. edwardsii* revealed significant genetic differentiation between populations of Australia (Tasmania; TAS) and New Zealand (NZ). This is consistent with previous investigations, suggesting that distance between Australia and New Zealand may influence neutral locus genetic structure and that local adaptation acting via adaptive divergence may influence outlier locus genetic structure within Australia and New Zealand (Morgan et al. 2013; Thomas and Bell 2013; Villacorta‐Rath et al. 2016). In the present work, the sample from TAS was highly differentiated from the NZ samples based on multiple test results, and had a higher *F*_IS_ value and a lower *A*_R_ value for both marker types than the mean values for the samples from NZ. Because the level of bidirectional gene flow between Australia and New Zealand is relatively low this means that national stocks are, to a large extent, independent of one another (i.e., there is no evidence of a source‐sink relationship). However, because in the present work the Australian context is only represented by one sample—from Tasmania—we need to be careful to not over‐interpret our findings at the international scale. Overall, our results are consistent with earlier work and do not support the hypothesis of a single genetic stock of *J. edwardsii* between Australia and New Zealand, despite the species' high potential for long‐distance pelagic larval dispersal.

#### 4.1.2 Genetic Structure Within New Zealand

Several earlier genetic studies of *J. edwardsii* have concluded that the species is a single panmictic stock across its austral range (Booth et al. 1990; Ovenden et al. 1992; Smith et al. 1980). However, panmixia within the NZ stock was not supported by a microsatellite‐based study describing finer‐scale population structure within New Zealand (Thomas and Bell 2013). Their study revealed a weak genetic signal delineating three subpopulations within NZ: a central NZ cluster (comprised of Wellington, Kaikoura, the southwest coast and the Chatham Islands), a northern NZ cluster (Hauraki Gulf) and a southern NZ cluster (represented by the Stewart Island population). For marine species, historical evidence of panmixia but more recent evidence of spatial differentiation is often reported and is most likely attributable to the increased resolving power of newer generations of molecular markers (e.g., allozymes versus RFLPs; microsatellites versus SNPs) and also to detailed sampling of more sites within a region and/or at areas of interest such as biogeographic boundaries (e.g., Apte and Gardner 2002; Wei et al. 2013a; Wenne 2023; Li et al. 2025).

Analyses of neutral SNP markers for rock lobsters from 22 New Zealand sites did not reveal pronounced patterns of population genetic structure. At best, they revealed subtle differentiation. The genetic divergence at site 902, as indicated by *F*_ST_ analyses, was not supported by individual‐based and spatial genetic analyses (STRUCTURE and GENELAND), nor by genetic indices (estimates of heterozygosity, inbreeding coefficient). The estimates of effective population size were all large or very large (as expected), providing no evidence of population reduction in terms of genetic diversity or size of the breeding population. Overall, the lack of pronounced genetic structure at the national scale detected by neutral markers suggests a high level of gene flow amongst all sites that has homogenised allele frequencies at neutrally evolving genetic loci (e.g., Hellberg et al. 2002) and very few, if any, natural barriers to gene flow. Such a finding is consistent with the long PLD of rock lobsters and also the findings of recent Lagrangian modelling of flow around coastal NZ, in particular when stepping stone sites are considered (Quigley et al. 2022, 2023; Chaput et al. 2023).

Outlier loci analyses identified three separate genetic groups within NZ: the Northwest (NW), Northeast (NE) and Southern (S) groups. This pattern of spatial differentiation for the outlier SNP loci is remarkably similar to the pattern of spatial differentiation described by Thomas and Bell (2013) based on their presumptively neutral microsatellite loci. Genetic differentiation of outlier loci can originate from local selection and adaptation (Seeb et al. 2011), which might be related to a single or a combination, of environmental factors. The identification of three distinct regional clusters provides supporting evidence that outlier loci are responding to geographically‐driven environmental variation, which in turn may lead to geographically unique genetic adaptations to local environmental conditions (e.g., Bourret et al. 2012; Segovia et al. 2017). The absence of genetic structuring or the presence of weak selection observed at neutral loci compared to strong differentiation at outlier or adaptive loci (which are usually defined a priori as those loci with high *F*_ST_ values) has been documented in various marine organisms characterised by high levels of gene flow (e.g., Krück et al. 2013; Candy et al. 2015; Poćwierz‐Kotus et al. 2015; Nayfa and Zenger 2016; Barth et al. 2017). For instance, in the case of Atlantic cod ( *Gadus morhua* ), adaptation to specific geographic or regional environmental conditions was elucidated by comparison to a reference genome. This enabled the mapping of selective markers to a large genomic region (~5 Mb) characterised by chromosomal rearrangement and a low recombination rate, suspected to contain genes crucial for survival at low salinities. Genomic islands of divergence occurring in regions of reduced recombination are one of the primary mechanisms driving adaptive divergence in local populations (Nosil and Feder 2012). While this mechanism may contribute to population differentiation in *J. edwardsii* , testing and confirmation awaits the availability of a reference genome for this species.

#### 4.1.3 Asymmetric Migration

The asymmetric migration pattern of S → NW → NE observed for *J. edwardsii* aligns with the predicted pattern of larval dispersal and settlement reported by Chiswell and Booth (2008, 2017). These models indicate that in New Zealand, most of the larvae released in the South Island settle in the North Island. Specifically, larvae released in the South Island tend to become entrained in the Southland Current, which flows up the east coast of the South Island. In the North Island, larvae released off North Cape are more likely to be entrained in the East Auckland Current, dispersing to the northeast of New Zealand, where they are entrained in the East Cape Current and then the Wairarapa Eddy. This pattern of asymmetric larval dispersal and connectivity is consistent with migration models based on empirical genetic data. For example, asymmetric larval dispersal from south to north was observed in modelling (particle tracking) studies of the New Zealand green‐lipped mussel, *P. canaliculus* (Quigley et al. 2023), as well as a study based on genetic data in the deep‐sea New Zealand lobster species (scampi), *Metanephros challengeri* (van der Reis et al. 2022). Understanding patterns of asymmetrical gene flow is now increasingly important for the management of fisheries species and also the conservation of foundational species (e.g., Xuereb et al. 2018; Mendiola and Ravago‐Gotanco 2021; Snead et al. 2023).

### 4.2 Seascape Genomics

#### 4.2.1 Neutral and Outlier Loci

For the neutral dataset, which is expected to reflect gene flow (and may therefore reflect natural barriers to gene flow) and not selection, GLM analysis of *F*_ST_ values revealed an association between environmental variation (tidal current speed [TC], roughness of the seafloor [ROUGH], depth [MLD] and sea surface temperature [SST]) and site‐specific genetic variation, whereas the BEST and dbRDA analyses did not reveal any significant influence of environmental variation on individual genetic variation. Thus, what evidence exists for the influence of environmental variation on neutral genetic variation is related to local habitat type/quality (i.e., TC, ROUGH, MLD) and also to sea water temperature (SST). Similar results have been reported for other lobster species, including the American lobster *Homarus americanus* (Lillis and Snelgrove 2010) and the European lobster *H. gammarus* (Galparsoro et al. 2009). In combination, these multi‐study results for three different lobster species indicate that characteristics of the physical environment such as presence of seafloor depressions and slopes (roughness), wave energy/bottom flow/tidal current speed and water depth, may all affect the neutral genetic variation of rock lobsters. The identification of the importance in the present study of SST is consistent with other studies of the ecological genetics of marine taxa that have also highlighted the importance of SST on influencing genetic variation (e.g., Selkoe et al. 2016). It seems most likely that gene flow (larval exchange) between populations in different regions is affected by this environmental variation, such that (semi‐permeable?) barriers to gene flow are created as a function of natural habitat patchiness, depth, water flow across the substratum and sea water temperature variation.

Analysis of the outlier dataset, which is usually assumed to reflect evidence of local selection, provided strong evidence of statistically significant results, but with few individual environmental variables being identified (GLM—CO_3_; DISTLM—ROUGH and SST). Seawater carbonate concentrations affect the calcification, structural integrity and growth of the crustacean exoskeleton (Feely et al. 2008; Taylor et al. 2015; Whiteley 2011). In other studies of rock lobsters, carbonate chemistry, related to reduced seawater pH, is associated with decreased somatic growth rate in the South African rock lobster *J. lalandii* (Knapp 2015) and high seawater temperature in relation to elevated partial pressure of carbon dioxide (*p*CO_2_) has been found to impact the growth and survival rates of *H. gammarus* larvae (Leiva et al. 2022). Thus, changes in seawater carbonate concentration and temperature are expected to have significant implications for the settlement, survival and fitness of *J. edwardsii* across NZ. These environmental changes likely play a role in the localised adaptation of *J. edwardsii* populations across its range, as documented for other marine organisms, both coastal and deep‐sea (Benestan et al. 2016; Bourret et al. 2024; Holland et al. 2022; Labrador et al. 2022; Mendiola and Ravago‐Gotanco 2021; Wei et al. 2013b; Zeng et al. 2020), highlighting the complex genotype‐environment association in marine ecosystems.

#### 4.2.2 Seascape Genomics—Challenges, Data Interpretation and the Real World

Seascape genomics is derived from landscape genomics and is a young discipline that is still developing (Liggins et al. 2013; Selkoe et al. 2016). Its main aim is to identify those environmental variables which explain patterns of spatial genetic variation, whether that be at neutral loci (e.g., as influenced by natural barriers to gene flow) and/or at outlier loci (e.g., as influenced by selection). One of the main challenges for seascape genomics is to be able to use a robust environmental data set that is composed of many different independent variables that are recorded at a spatial (and sometimes temporal) scale that is biologically relevant. Here, we were able to use a multivariate environmental data set newly developed for the New Zealand Exclusive Economic Zone (NZ EEZ) by the National Institute of Water & Atmospheric Research (NIWA—http://www.niwa.co.nz). Whilst the different environmental variables are resolved at different spatial scales (Table S1), for a long‐lived organism such as *J. edwardsii* with an extremely long PLD, these spatial scales are fine enough to be biologically relevant. Management decisions should only be made when based on the best data available, and right now the new environmental data set for the NZ EEZ is of high quality (numerous environmental variables are included and its spatial resolution is high) and without doubt the best that is presently available for the country's EEZ. The question then arises about which environmental variables to include in the analyses and how best to incorporate geospatial alongside environmental variables. Here, following published examples, we have taken a moderately stringent approach (pairs of variables with *R* > 0.8 are reduced to a single representative variable) and then independent variables with VIF > 10.0 are excluded. These criteria are arbitrary but widely used (e.g., Silva and Gardner 2016; Zeng et al. 2020; Holland et al. 2022), and it has long been recognised that different inclusion/rejection values will provide different results because more or less stringent criteria change the number of variables in the model. Thus, the aim is to achieve a balance between being too stringent (excluding too many environmental variables) and being too lenient (including too many variables). Here, we reduced our environmental data set from 34 to 16 variables, and we also dropped all three geospatial variables in favour of three correlated environmental variables. There is no single best way to analyse seascape genomics data and, as expected, each type of analysis has strengths and weaknesses (Rellstab et al. 2015), although redundancy analysis (as employed here by us) has been shown to ‘… accommodate the genomic and environmental complexity found in nature, producing a powerful and efficient tool for landscape genomics’ (page 2298 in Capblancq and Forester 2021). Our combined analyses provide results with key explanatory environmental variables that can be interpreted by managers, both in terms of neutral loci and gene flow responding to natural barriers to movement and also outlier loci and regional (spatial) divergence that may result in uniquely adapted genomes.

### 4.3 Information Content of Neutral Versus Outlier SNP Loci

While spatially‐explicit genetic structure attributed to local adaptation should not influence putatively neutral markers, a phenomenon known as Isolation by Adaptation (IBA) at neutral loci may contribute to the subtle pattern of genetic variation that we observed (site 902 being different from all other NZ sites). Essentially, selective pressure can translate into divergence at non‐linked neutral loci (e.g., Ólafsdóttir et al. 2007; Nosil et al. 2008). Several mechanisms of how selective pressure might translate into divergence at neutral loci have been described. One is genetic hitchhiking—a phenomenon where the frequency of neutral loci can be increased by a selective force if they are linked to a locus experiencing selection (Bierne 2010; Roesti et al. 2014). The hitchhiking effect on neutral loci reduces with distance to that gene/locus under selection; however, this effect can extend for a considerable distance. Simulations have shown that strong selection can promote divergence at neutral loci as far apart as the length of the entire chromosome (Charlesworth et al. 1997; Kim and Stephan 2002). The hitchhiking effect strongly depends on the recombination frequency:selection strength ratio due to the chance of the bond between neutral and selected loci being broken by recombination (Barton 2000; Andolfatto 2001; Kim and Stephan 2002). Another mechanism that might explain the observed pattern of differentiation is when a marker is located in a region already under selection in the population. In such cases, this neutral marker is likely to show a substantial level of divergence (Nosil et al. 2009; Gagnaire et al. 2015). Supporting this scenario is the ‘genomic island growth’ hypothesis, which predicts that regions of genetic differentiation will cluster in proximity to one another rather than being randomly distributed throughout the genome (Gavrilets 2004; Kirkpatrick and Barton 2006). In most cases, genomic islands of divergence are associated with centromeric, telomeric or rearranged regions of the chromosome where the recombination rate is reduced due to a lower chance of forming a chiasma (Cruickshank and Hahn 2014; Turner et al. 2005).

Divergent selection may affect completely non‐linked neutral loci and promote their divergent differentiation by reducing the effective population size (*N*_e_) and increasing the effect of genetic drift in subpopulations (Nosil et al. 2005, 2009). Our estimates of *N*_e_ were all high or very high and do not, therefore, provide support for the idea of divergent differentiation. Regardless, if it occurs, local selection effectively reduces gene flow by causing a lower level of migration because migrants are selected against if they do not possess the advantageous alleles for survival in the new environment (Charlesworth et al. 1997). For *J. edwardsii* , local adaptive divergence across New Zealand may create genetic barriers affecting settlement and post‐settlement mortality for migrating individuals (almost exclusively larvae). This could potentially pose a threat to the species' abundance as reduced gene flow limits genetic exchange and hinders adaptation to changing environmental conditions.

### 4.4 Management Implications

Analyses of neutral SNP loci confirm earlier suggestions of high levels of gene flow and very little, if any, spatial population differentiation within New Zealand. These results support the present Quota Management Areas (QMAs) for this species in New Zealand (https://www.mpi.govt.nz/dmsdocument/26779/direct/), in as much as we could not identify source or sink populations that are unsupported by the QMAs. However, analyses of the outlier SNP loci support the existence of three regional stocks of rock lobsters in New Zealand, which may be attributed to selection based on regional environmental differences. As such, the outlier SNP analyses may help with future planning for rock lobster management as data from loci under selection is now increasingly being taken into account for management purposes. For example, information from outlier SNP loci may be used by the New Zealand government (FisheriesNZ as part of the Ministry for Primary Industries) and/or by the industry itself (New Zealand Rock Lobster Industry Council) to better manage different stocks based on environmental variables to help preserve unique adaptive genetic variation (e.g., Russello et al. 2012; Taillebois et al. 2021). Our results also highlight the role that habitat characteristics such as carbonate concentration and sea surface temperature may have for the long‐term survival and adaptability of this species under future climate change scenarios. Finally, over the last 20 to 30 years, consideration has often been given to the establishment of new rock lobster aquaculture initiatives in New Zealand, given the market value of individual animals. However, costs associated with development (e.g., infrastructure) and permitting (e.g., catching larvae from the wild and transferring them to the facility) have historically counted against new ventures. Nonetheless, both the population and the seascape genomics results reported here may help support aquaculture development initiatives (e.g., selective breeding programmes, similar to those described for the ornate spiny lobster, *Panulirus ornatus* —Farhadi et al. 2022), in the future, if and when New Zealand develops a rock lobster aquaculture industry.

## Funding

This research was funded in part by Seafood Innovations Limited (Wellington, New Zealand) and the New Zealand Rock Lobster Industry Council. I.I. was supported by a Victoria Doctoral Scholarship and Kathleen Stewart Postgraduate Award. J.M.S., N.M. and J.J.B. were supported by an Australian Research Council Discovery Project (Project no. DP150101491). J.P.A.G. and M.J.R.M., as part of The Moana Project (www.moanaproject.org), were funded by the New Zealand Ministry of Business Innovation and Employment (MBIE), contract number METO1801.

## Conflicts of Interest

The authors declare no conflicts of interest.

## Supporting information


**Figure S1:** Multicollinearity plot showing pairwise correlations amongst 34 environmental variables (before testing for independence) collected across various sites in New Zealand. Negative correlations are depicted in red, while positive correlations are represented in blue. The intensity of colour and the size of the circles correspond to the correlation coefficients.
**Figure S2:** Multicollinearity plot showing pairwise correlations amongst 16 environmental variables (after testing for independence) collected across various sites in New Zealand. Negative correlations are depicted in red, while positive correlations are represented in blue. The intensity of colour and the size of the circles correspond to the correlation coefficients.
**Figure S3:** Plots of the posterior distribution across all loci for the analysis of the different Migrate‐n models.
**Table S1:** List of 34 environmental variables collected across various sites in New Zealand.
**Table S2:** Variance inflation factor values of 16 environmental variables (from an initial 34 variables—Table S1) retained for seascape genomics analysis.

## Data Availability

The final SNP dataset, including both outlier and neutral loci in genepop format, along with the R Markdown file detailing most of the analyses, will be deposited in Dryad or a similar open‐access free‐of‐cost data repository on publication of this manuscript.
