# Supplementary material for: High Gene Flow With Patterns of Asymmetric Connectivity and Adaptive Divergence in the New Zealand Southern Rock Lobster, Jasus edwardsii (Hutton, 1875)
Source: Evol Appl. 2026 Apr 7;19(4):e70233. doi: 10.1111/eva.70233 (PMC13054955; doi:10.1111/eva.70233)
Supplement: Supplementary file 1 — Figure S1: Multicollinearity plot showing pairwise correlations amongst 34 environmental variables (before testing for independence) collected across various sites in New Zealand. Negative correlations are depicted in red, while positive correlations are represented in blue. The intensity of colour and the size of the circles correspond to the correlation coefficients. Figure S2: Multicollinearity plot showing pairwise correlations amongst 16 environmental variables (after testing for independence) collected across various sites in New Zealand. Negative correlations are depicted in red, while positive correlations are represented in blue. The intensity of colour and the size of the circles correspond to the correlation coefficients. Figure S3: Plots of the posterior distribution across all loci for the analysis of the different Migrate‐n models. Table S1: List of 34 environmental variables collected across various sites in New Zealand. Table S2: Variance inflation factor values of 16 environmental variables (from an initial 34 variables—Table S1) retained for seascape genomics analysis. [file EVA-19-e70233-s001.docx]

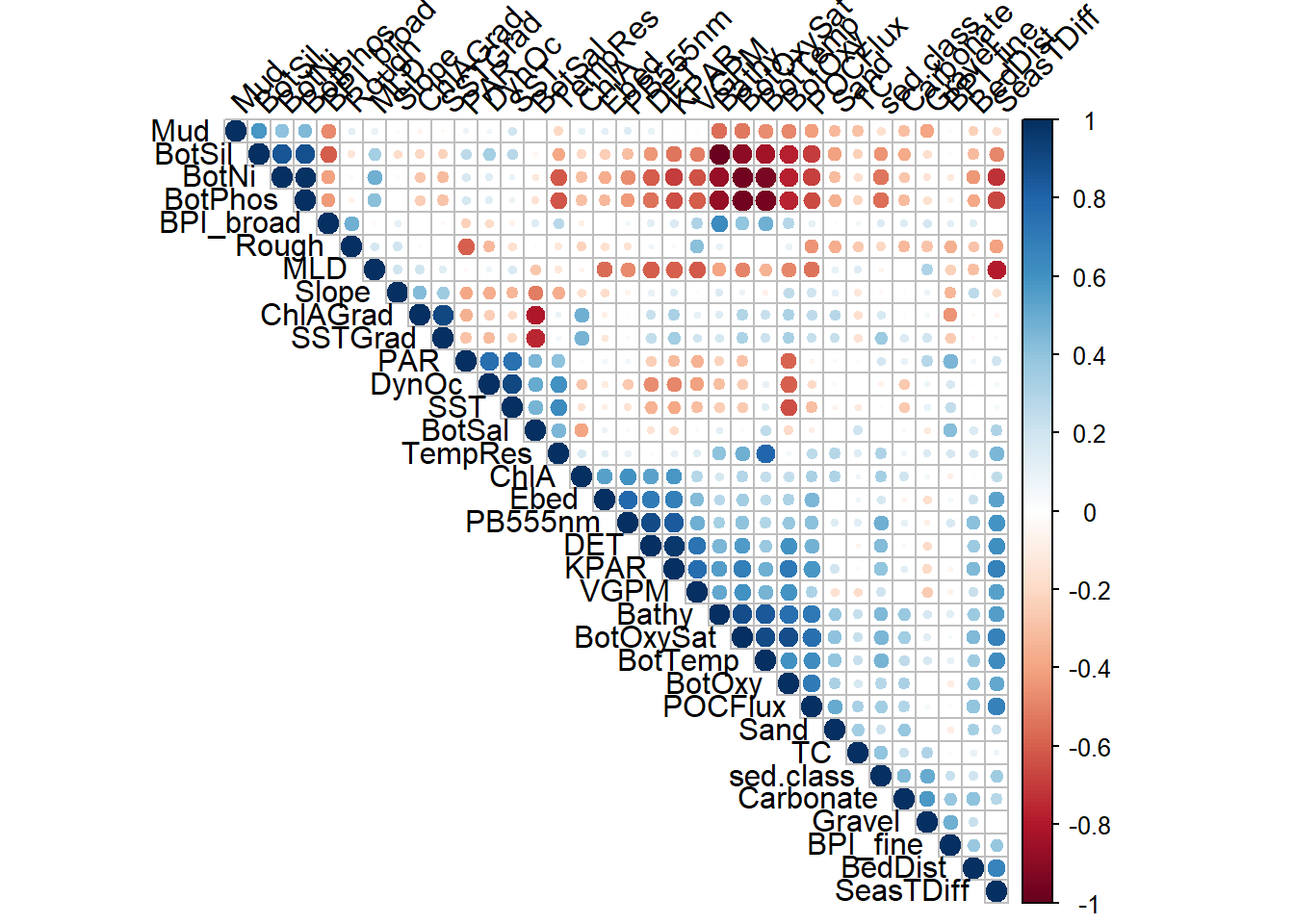


**FIGURE S1** Multicollinearity plot showing pairwise correlations amongst 34 environmental variables (before testing for independence) collected across various sites in New Zealand. Negative correlations are depicted in red, while positive correlations are represented in blue. The intensity of colour and the size of the circles correspond to the correlation coefficients.


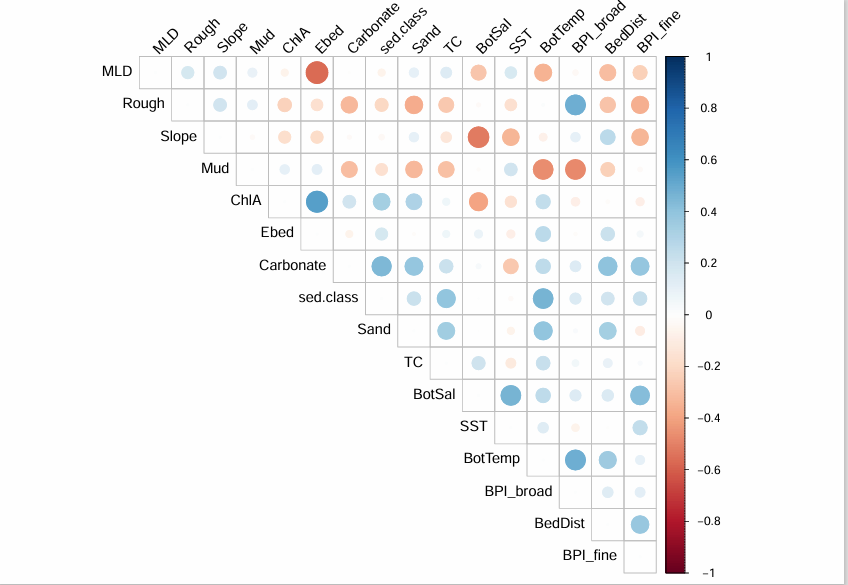


**FIGURE S2** Multicollinearity plot showing pairwise correlations amongst 16 environmental variables (after testing for independence) collected across various sites in New Zealand. Negative correlations are depicted in red, while positive correlations are represented in blue. The intensity of colour and the size of the circles correspond to the correlation coefficients.


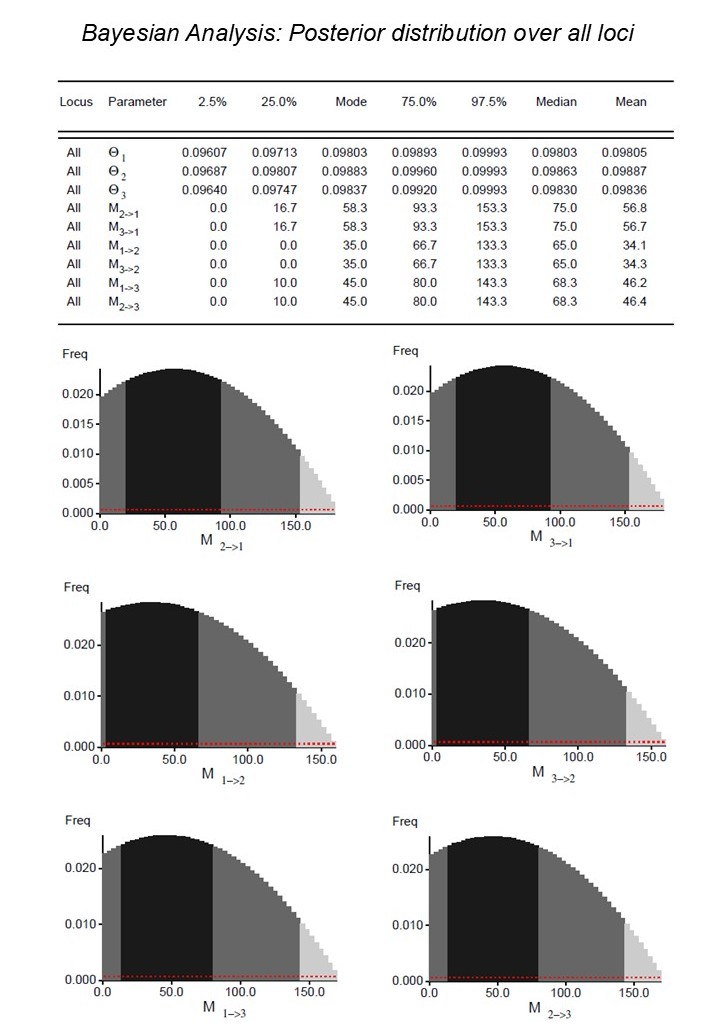


**Figure S3** Plots of the posterior distribution across all loci for the analysis of the different Migrate-n models.

**Table S1** List of 34 environmental variables collected across various sites in New Zealand.

| **Abbreviation** | **Full name** | **Projection** | **Temporal range** | **Description** | **Native Resolution** | **method for modifying resolution** | **Units** | **Source** |
| --- | --- | --- | --- | --- | --- | --- | --- | --- |
| **Bathy** | Bathymetry | Custom Alber Equal Area (central meridian: 175; standard parallel 1: -30; standard parallel 2: -50; standard parallel 3: -40; geographic datum: WGS 1984) | Static | Depth at the seafloor was interpolated from contours generated from various sources, including multi-beam and single-beam echo sounders, satellite gravimetric inversion, and others (Mitchell et al., 2012). | 250 m | Cubic interpolation —determines the new value of a cell based on fitting a smooth curve through the 16 nearest input cell centres. (Note there are 16 x 250m cells in a 1km grid) | m | Mitchell, J.S., Mackay, K.A., Neil, H.L., Mackay, E.J., Pallentin, A., Notman P., 2012. Undersea New Zealand, 1:5,000,000. NIWA Chart, Miscellaneous Series No. 92. Available at: https://niwa.co.nz/our-science/oceans/bathymetry/download-the-data (accessed: 29/10/2019) |
| **BPI_fine** | BPI_fine | Custom Alber Equal Area | Static | Terrain metrics were calculated using an inner annulus of 2 km and a radius of 12 km using the NIWA bathymetry layer in the Benthic Terrain Modeler in ArcGIS 10.3.1.1 (Wright et al. 2012). Bathymetric Position Index (BPI) is a measure of where a referenced location is relative to the locations surrounding it. | 250 m | Cubic interpolation —determines the new value of a cell based on fitting a smooth curve through the 16 nearest input cell centres. (Note there are 16 x 250m cells in a 1km grid) | m | NIWA, unpublished |
| **BPI_broad** | BPI_broad | Custom Alber Equal Area | Static | Terrain metrics were calculated using an inner annulus of 12 km and a radius of 62 km using the NIWA bathymetry layer in the Benthic Terrain Modeler in ArcGIS 10.3.1.1 (Wright et al. 2012). Bathymetric Position Index (BPI) is a measure of where a referenced location is relative to the locations surrounding it. | 250 m | Cubic interpolation —determines the new value of a cell based on fitting a smooth curve through the 16 nearest input cell centres. (Note there are 16 x 250m cells in a 1km grid) | m | NIWA, unpublished |
| **Rough** | Roughness | Custom Alber Equal Area | Static | Roughness of the seafloor calculated as the standard deviation of depths in a surrounding 3 x 3 km neighbourhood (Leathwick et al., 2012). Terrain Ruggedness (VRM) as the variation in three-dimensional orientation of grid cells within a neighbourhood. Vector analysis is used to calculate the dispersion of vectors normal (orthogonal) to grid cells within the specified neighbourhood | 250 m | Cubic interpolation —determines the new value of a cell based on fitting a smooth curve through the 16 nearest input cell centres. (Note there are 16 x 250m cells in a 1km grid) | m | Leathwick et al. (2012) |
| **Slope** | Slope | Custom Alber Equal Area | Static | Bathymetric slope was calculated from bathymetric depth and is the degree change from one depth value to the next. | z-value | Cubic interpolation —determines the new value of a cell based on fitting a smooth curve through the 16 nearest input cell centres. (Note there are 16 x 250m cells in a 1km grid) | Degree | NIWA, unpublished |
| **Beddist** | Benthic sediment disturbance | Custom Alber Equal Area | 1/7/2017-30/6/2018 | One-year mean value of friction velocity derived from (1) hourly estimates of surface wave statistics (significant wave height, peak wave period) from outputs of the NZWAVE_NZLAM wave forecast, at 8-km resolution, (2) median grain size (d50), at 250 m resolution, (3) water depth, at 25-m resolution. Benthic sediment disturbance from wave action was assumed to be zero where depth ≥ 200m. | 250 m | Bilinear interpolation was used to interpolate surface wave statistics (significant wave height, peak wave period) from the 8 km resolution wave model grid to the output grid. For points less than 25 km from shore, the interpolated significant wave height was corrected by a scaling factor dependent on fetch in the upwave direction. | ms-1 | [https://one.niwa.co.nz/display/WRH/Implementation+of+globalwave-17%2C+nzwave-8%2C+nzwave-2/;  Swart, D. H. (1974), Offshore sediment transport and equilibrium beach profiles, Delft Hydraul. Lab. Publ. 131, Delft.](https://one.niwa.co.nz/display/WRH/Implementation+of+globalwave-17%2C+nzwave-8%2C+nzwave-2/;Swart,%20D.%20H.%20(1974),%20Offshore%20sediment%20transport%20and%20equilibrium%20beach%20profiles,%20Delft%20Hydraul.%20Lab.%20Publ.%20131,%20Delft.) |
| **BotNi** | Bottom nitrate | Custom Alber Equal Area | Static | Annual average water nitrate concentration at the seafloor (using NZ bathymetry layer) based on methods from Dunn et al. 2002. Oceanographic data from CARS2009 (2011). | approx 41 km (1/2 degree) | beta spline interpolation (model the relationship and fit this to the 250m depth data) | umol l-1 | NIWA, unpublished |
| **BotOxy** | Dissolved oxygen at depth | Custom Alber Equal Area | Static | Annual average water oxygen concentration at the seafloor (using NZ bathymetry layer) based on methods from Dunn et al. 2002. Oceanographic data from CARS2009 (2011). | approx 41 km (1/2 degree) | Beta spline | ml l-1 | NIWA, unpublished |
| **BotOxySat *** | Oxygen saturation at depth | Custom Alber Equal Area | Static | Annual average oxygen saturation at the depths | approx 41 km (1/2 degree) | Beta spline | umol l-1 | NIWA, unpublished |
| **BotPhos** | Bottom phosphate | Custom Alber Equal Area | Static | Annual average water phosphate concentration at the seafloor (using NZ bathymetry layer) based on methods from Dunn et al. 2002. Oceanographic data from CARS2009 (2011). | approx 41 km (1/2 degree) | Beta spline | umol l-1 | NIWA, unpublished |
| **BotSal** | Salinity at depth | Custom Alber Equal Area | Static | Annual average water salinity concentration at the seafloor (using NZ bathymetry layer) based on methods from Dunn et al. 2002. Oceanographic data from CARS2009 (2011). | approx 41 km (1/2 degree) | Beta spline | psu | NIWA, unpublished |
| **BotSil** | Bottom silicate | Custom Alber Equal Area | Static | Annual average water silicate concentration at the seafloor (using NZ bathymetry layer) based on methods from Dunn et al. 2002. Oceanographic data from CARS2009 (2011). | approx 41 km (1/2 degree) | Beta spline | umol l-1 | NIWA, unpublished |
| **BotTemp** | Temperature at depth | Custom Alber Equal Area | Static | Annual average water temperature at the seafloor (using NZ bathymetry layer) based on methods from Ridgway et al. (2002). The oceanographic data used to generate these climatological maps were computed by objective analysis of all scientifically quality-controlled historical data from the Commonwealth Scientific and Industrial Research Organisation (CSIRO) Atlas of Regional Seas database (CARS2009). | approx 41 km (1/2 degree) | Beta spline | °C km-1 | NIWA, unpublished |
| **carbonate** | Percent gravel | Custom Alber Equal Area | Static | The percent carbonate layers for the region were developed from >30,000 raw sediment sample data compiled in dbseabed (Jenkins et al. 1997), which were then imported into ArcGIS and interpolated using Inverse Distance Weighting (Bostock et al. 2018) | 1 km | bi-linear interpolation | % | H Bostock et al., 2018 |
| **Chl-a** | Chlorophyll-a concentration | Custom Alber Equal Area | July 2002 – March 2019 | A proxy for the biomass of phytoplankton present in the surface ocean (to ~30 m). Blended from a coastal Chl-a estimate (quasi-analytic algorithm (QAA), local a_ph_*(555)) and the default open-ocean chl-a value from MODIS-Aqua (v2018.0). | 4 km (ocean) 500 m (coastal) | Nearest-neighbour 500 m to to 1 km; otherwise, bilinear interpolation | mg m^-3^ | NIWA unpublished; Based on processing described in Pinkerton et al. (2018) and updated in Pinkerton et al. (2020). QAA algorithm detailed in Lee et al., 2002, v5 update, Lee et al. 2009 |
| **Chl-a.Grad** | Chlorophyll-a concentration spatial gradient | Custom Alber Equal Area | July 2002 – March 2019 | Smoothed magnitude of the spatial gradient of annual mean Chl-a. A product that has started to appear in papers as a possible indicator of the distribution of zooplankton and mesopelagic fish. Derived from Chl-a described above. | 500 m | Nearest-neighbour to 1 km;  Bilinear interpolation to 250 m | mg/m^3^/km | NIWA unpublished; Based on processing described in Pinkerton et al. (2018) |
| **DET** | Detrital absorption | Custom Alber Equal Area | July 2002 – March 2019 | Total detrital absorption coefficient at 443 nm, including due to coloured dissolved organic matter (CDOM) and particulate detrital absorption. Estimated using quasi-analytic algorithm (QAA) applied to MODIS-Aqua data, blended with *adg_443_giop* ocean product (Werdell, 2019). | 4 km (ocean) 500 m (coastal) | Nearest-neighbour 500 m to to 1 km; otherwise, bilinear interpolation | m^-1^ | NIWA unpublished; Based on processing described in Pinkerton et al. (2018). Processing for *adg_443_giop* ocean product described in Werdell (2019). |
| **DynOc** | Dynamic oceanography | Custom Alber Equal Area |  | Mean of the 1993-1999 period sea surface above geoid, corrected from geophysical effects taken for the NZ region. This broadly corresponds to mean surface velocity recorded from drifters in the NZ region (Hadfield pers comm). | 250 m | cubic interpolation | m | NIWA, unpublished |
| **Gravel** | Percent gravel | Custom Alber Equal Area | Static | The percent gravel layers for the region were developed from >30,000 raw sediment sample data compiled in dbseabed (Jenkins et al. 1997), which were then imported into ArcGIS and interpolated using Inverse Distance Weighting (Bostock, pers comm) | 1km | bi-linear interpolation | % | H Bostock et al., 2018 |
| **K_par_** | Diffuse downwelling attenuation | Custom Alber Equal Area | July 2002 – March 2019 | vertical attenuation of diffuse, downwelling broadband irradiance (Photosynthetically Available Radiation, PAR, 400–700 nm). Merged coastal and open-ocean product based on MODIS-Aqua data. Coastal: estimated from inherent optical properties (QAA). Ocean: estimated from K_490_ using Morel et al. (2007) | 4 km (ocean) 500 m (coastal) | Nearest-neighbour 500 m to to 1 km; otherwise, bilinear interpolation | m^-1^ | NIWA unpublished; Based on processing described in Pinkerton et al. (2018) |
| **MLD** | Mixed layer depth | Custom Alber Equal Area | July 2002 – March 2019 | The depth that separates the homogenized mixed water above from the denser stratified water below. Based on GLBu0.08 hindcast results using a potential density difference of 0.030 kg m^-3^ from the surface. Models used are: (1) hycom: from day 265 (2008) to present; (2) fnmoc: from day 169 (2005) to present; (3) soda: from day 249 (1997) to end of 2004; (4) tops: from day 001 (2005) to 225 (2010). | 9 km | Bilinear interpolation | m | Metzger et al. (2007); Chassignet et al. (2007); Wallcraft et al. (2009). Data: orca.science.oregonstate.edu |
| **Mud** | Percent mud | Custom Alber Equal Area | Static | The percent mud layers for the region were developed from >30,000 raw sediment sample data compiled in dbseabed (Jenkins et al. 1997), which were then imported into ArcGIS and interpolated using Inverse Distance Weighting (Bostock, pers comm) | 1km | bi-linear interpolation | % | H Bostock et al., 2018 |
| **sand** | sand | Custom Alber Equal Area | Static | The percent sand layers for the region were developed from >30,000 raw sediment sample data compiled in dbseabed (Jenkins et al. 1997), which were then imported into ArcGIS and interpolated using Inverse Distance Weighting (Bostock, pers comm) | 1km | bi-linear interpolation | % | H Bostock et al., 2018 |
| **SeasTDiff** | Annual amplitude of sea floor temperature | Custom Alber Equal Area |  | Smoothed difference in seafloor temperature between the three warmest and coldest months. Providing a measure of temperature amplitude through the year. | 250 km | cubic interpolation | °C km-1 | NIWA, unpublished data |
| **SST** | Sea surface temperature | Custom Alber Equal Area | 1981-2018 (ocean) 2002-2018 (coastal) | Blended from OI-SST (Reynolds et al., 2002) ocean product and MODIS-Aqua SST coastal product. Long-term (2002–2017) average values at 250 m resolution. | 0.25° (ocean) 1 km (coastal) | Bilinear interpolation | °C | NIWA unpublished; Coastal based on processing described in Pinkerton et al. (2018). Ocean: Reynolds et al., (2002) |
| **SstGrad** | Sea surface temperature gradient | Custom Alber Equal Area | 1981-2018 (ocean) 2002-2018 (coastal) | Smoothed magnitude of the spatial gradient of annual mean SST. This indicates locations in which frontal mixing of different water bodies is occurring (Leathwick et al., 2006). Derived from SST described above at two resolutions and merged. | 0.25° (ocean) 1 km (coastal) | Bilinear interpolation | °C km^-1^ | NIWA unpublished |
| **SuspPM** | Suspended particulate matter | Custom Alber Equal Area |  | Indicative of total suspended particulate matter concentration. Based on SeaWiFS ocean colour remote sensing data (Pinkerton & Richardson 2005); modified Case 2 atmospheric correction (Lavender et al. 2005); modified Case 2 inherent optical property algorithm (Pinkerton et al. 2006) | 4km |  | Indicative of total suspended particulate matter concentration (g m-3) | Pinkerton (2016) |
| **TC** | Tidal Current speed | Custom Alber Equal Area | N/A | Maximum depth-averaged (NZ bathymetry) flows from tidal currents calculated from a tidal model for New Zealand waters (Walters et al., 2001) | 250 m | Tidal constituents (magnitude A and phase phi, represented as real and imaginary parts X + iY = A*exp(i*phi)) for sea surface height and currents (8 components) were taken from the EEZ tidal model, on an unstructured mesh at variable spatial resolution. The complex components were bilinearly interpolated to the output grid. | ms-1 | Walters, R.A., Goring, D.G., Bell, R.G. (2001) Ocean tides around New Zealand. New Zealand Journal of Marine and Freshwater Research, 35: 567-579. |
| **TempRes** | Temperature residuals | Custom Alber Equal Area |  | Residuals from a GLM relating temperature to depth using natural splines – this highlights areas where average temperature is higher or lower than would be expected for any given depth | 250 m | cubic interpolation | °C | Leathwick et al. (2006) |
| **PB** | Particulate backscatter at 555 nm (previously used to generate 'turbidity') | Custom Alber Equal Area | July 2002 – March 2019 | Optical particulate backscatter at 555 nm estimated using blended coastal and ocean products. Coastal: QAA v5 product bbp555 from MODIS-Aqua data. Ocean: *bbp_555_giop* ocean product (Werdell, 2019). Result calculated as long-term (2002–2017) average. | 4 km (ocean) 500 m (coastal) | Bilinear interpolation | m^-1^ | NIWA unpublished; Based on processing described in Pinkerton et al. (2018). Processing for *bbp_555_giop* ocean product described in Werdell (2019). |
| **Ebed** | Seabed incident irradiance | Custom Alber Equal Area | July 2002 – March 2019 | Broadband (400–700 nm) incident irradiance (E/m^2^/d) at the seabed, averaged over a whole year. Estimated by combining incident irradiance at the sea surface (Frouin et al., 2012; this table), diffuse downwelling irradiance attenuation (K_PAR_; this table) and bathymetric depth at monthly resolution. Derived from blended coastal (QAA) and open-ocean attenuation products. | 4 km (ocean); 500 m (coastal) | Nearest-neighbour 500 m to to 1 km; otherwise, bilinear interpolation | E/m^2^/d | NIWA unpublished, based on processing described in Pinkerton et al. (2018) |
| **VGPM** | Net primary production by the vertically-generalised production model | Custom Alber Equal Area | July 2002 – March 2019 | Daily production of organic matter by the growth of phytoplankton in the surface mixed layer, net of phytoplankton respiration. Estimated at monthly resolution based on satellite observations of chl-a, PAR and SST, and model-derived estimates of mixed-layer depth, using the vertically-generalised production model (Behrenfeld & Falkowski, 1997). | 9 km | Bilinear interpolation | mgC/m^2^/d | Behrenfeld & Falkowski (1997) |
| **fluxSeabed (POCFlux)** | Downward vertical flux of particulate organic matter at the seabed | Custom Alber Equal Area | July 2002 – March 2019 | Net primary production in the surface mixed layer estimated as the VGPM model (Behrenfeld & Falkowski 1997; this table). Export fraction and flux attenuation factor with depth estimated by refitting sediment trap and thorium-based measurements to environmental data (VGPM, SST) as Lutz et al. (2002), Pinkerton et al. (2016) and using data from Cael et al. (2017). | 9 km | Bilinear interpolation | mgC/m2/d | NIWA unpublished. Based on processing described in Pinkerton et al. (2002) with new data from Cael et al. (2017). |
| **PAR** | Photo-synthetically active radiation | Custom Alber Equal Area | July 2002 – March 2019 | Daily-integrated, broadband, incident irradiance at the sea-surface based on day length, solar elevation and measurements of cloud cover from ocean colour satellites (Frouin et al., 2012). | 4 km | Bilinear interpolation | Einsteins m^-2^ d^-1^ | Frouin et al. (2012) |

**TABLE S2** Variance inflation factor values of 16 environmental variables (from an initial 34 variables – Table S1) retained for seascape genomics analysis.

| **Variable** | **VIF** |
| --- | --- |
| BedDist | 2.69 |
| BotSal | 7.21 |
| BotTemp | 5.07 |
| BPI_Broad | 3.44 |
| BPI_fine | 3.01 |
| ChlA | 3.29 |
| Carbonate | 6.59 |
| Ebed | 5.93 |
| MLD | 5.54 |
| Mud | 3.32 |
| Rough | 6.55 |
| Sand | 3.15 |
| Sed.class | 2.09 |
| Slope | 3.95 |
| SST | 5.69 |
| TC | 3.01 |
